# The Dark Side of Lead-Free Metal Halide Nanocrystals: Substituent-Modulated Photocatalytic Activity in Benzyl Bromide Reduction

**DOI:** 10.1021/acsenergylett.3c00771

**Published:** 2023-05-30

**Authors:** Ignacio Rosa-Pardo, Dongxu Zhu, Alejandro Cortés-Villena, Mirko Prato, Luca De Trizio, Liberato Manna, Raquel E. Galian, Julia Pérez-Prieto

**Affiliations:** † Institute of Molecular Science, University of Valencia, c/Cat. José Beltrán 2, Paterna, 46980 Valencia, Spain; ‡ Nanochemistry, Istituto Italiano di Tecnologia, Via Morego 30, 16163 Genova, Italy; § Materials Characterization Facility, Istituto Italiano di Tecnologia, Via Morego 30, 16163 Genova, Italy

## Abstract

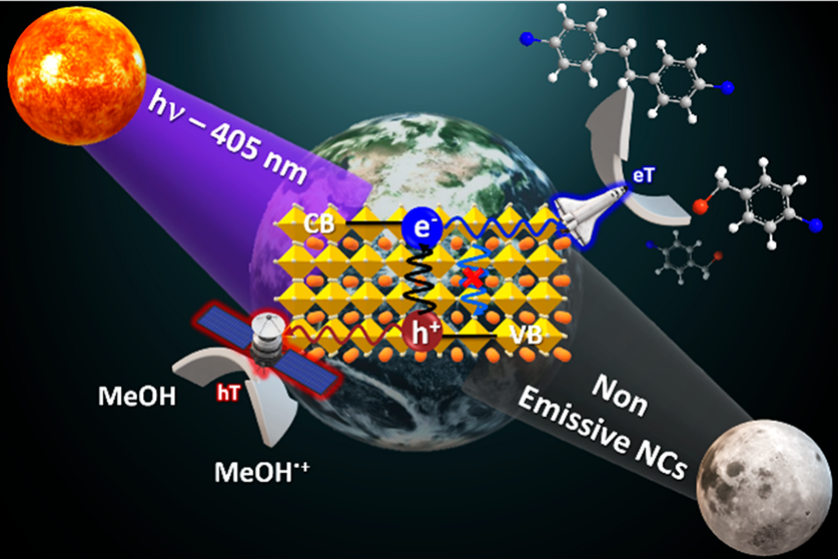

We illustrate here the high photocatalytic activity of sustainable lead-free metal halide nanocrystals (NCs), namely, Cs_3_Sb_2_Br_9_ NCs, in the reduction of *p*-substituted benzyl bromides in the absence of a cocatalyst. The electronic properties of the benzyl bromide substituents and the substrate affinity to the NC surface determine the selectivity in C–C homocoupling under visible light irradiation. This photocatalyst can be reused for at least three cycles and preserves its good performance with a turnover number of ca. 105,000.

Photosynthesis is a natural process that plants exploit to produce sugars from light, water, and CO_2_. This reaction allows plants to store solar energy in the form of chemical bonds.^1^ Mimicking photosynthesis in the laboratory to produce valuable chemicals from sunlight represents a promising way to exploit renewable green energy. In this regard, the synthesis of organic molecules via photoredox catalysis has been traditionally performed with noble metal catalysts, which are characterized by high cost, difficult recyclability, and complex synthetic preparation.^2−4^ On this matter, the development of a new generation of efficient photocatalysts with easy and low-cost preparation, good performance, durability, and reusability is required to accomplish photocatalyzed reactions on complex molecules.^5,6^ Semiconductor nanocrystals (NCs), such as metal chalcogenides and the more recent halide perovskites, have emerged as very promising candidates for photocatalysis since they can efficiently harvest photons. The electrons in the conduction band (CB) and holes in the valence band (VB) can promote reduction and oxidation, respectively.^7^ In detail, lead halide perovskite NCs (APbX_3,_ A = CH_3_NH_3_^+^, Cs^+^; X = Cl^–^, Br^–^, I^–^) have been spotted as optimal photocatalysts (for solar energy capture^8^) thanks to their appealing optoelectronic properties, such as a strong absorption in the UV–visible range, long excited-state lifetimes, effective charge separation and transport, and ultrafast charge transfer processes,^9−14^ together with their simple preparation from earth-abundant materials. Moreover, the photogenerated charges are trapped at different surface sites to conduct redox reactions with electron acceptors and donors.^15^ Indeed, APbX_3_ NCs have been explored as photocatalysts in the oxidative degradation of photosensitizers,^16^ CO_2_ reduction,^17,18^ and organic transformations, such as alcohol oxidation,^19^ C(sp^3^)-H activation,^20^ oxidative palladium Suzuki coupling reactions,^21^ and C–C, C–O, and C–N bond formation.^22^ In particular, the Csp^3^–Csp^3^ bond formation via C–H activation of aldehydes by CsPbBr_3_ NCs required the consumption of a cocatalyst and a base to drive forward the reaction.^22,23^ In this regard, we have recently demonstrated the suitability of colloidal CsPbBr_3_ nanocubes (size of ca. 11 nm) as photosensitizers for a demanding photoredox catalytic homo- and cross-coupling of benzyl bromides at room temperature by using visible light and *N*,*N*-diisopropylethylamine as the electron donor, without requiring the presence of a cocatalyst and a base.^24^ The cooperative action between the NC surface and the molecules constituting their ligand shell was crucial to make this reaction thermodynamically favorable for the effective synthesis of bibenzyl derivatives. It is noteworthy that the photocatalytic preparation of bibenzyl molecules can be very precious for the pharmaceutical industry, since these moieties are present in a wide variety of dihydrostilbenoids which form/constitute several antitumor and cardiovascular protective drugs.^25−27^ However, after one photocatalytic cycle, the loss of surfactants during the cleaning of the NCs led to their transformation into micrometer-sized particles, thereby significantly reducing their photocatalytic activity.^24^ In addition, the employment of toxic lead greatly limits their practical applications.

Lead-free metal halide materials, such as Bi- and Sb-based NCs, having an A_3_B_2_X_9_ (A = CH_3_NH_3_^+^, Cs^+^; B = Sb^3+^ or Bi^3+^, X = Cl^–^, Br^–^, I^–^) stoichiometry, have been proposed as good candidates for heterogeneous photocatalysis thanks to their higher chemical stability and better performance than those of Pb-based perovskite NCs.^28,29^ These compounds crystallize in a 2D layered phase (space group *P*3̅*m*1), which can be thought as resulting from the replacement of Pb^2+^ cations by trivalent B^3+^ cations, requiring the removal of every third Bi or Sb layer along the (111) plane of the 3D parent perovskite structure to maintain charge neutrality.^30^ Moreover, Cs_3_Sb_2_Br_9_ and Cs_3_Bi_2_Br_9_ present a direct band gap transition which enables an efficient absorption of photons to induce photocatalytic reactions.^31,32^ These materials have already become one of the most promising alternatives for optoelectronic and photocatalytic applications due to their interesting photoredox properties (band gap of 2.46 and 2.56 eV, and conduction band of −2.98 and −3.03 eV for Cs_3_Sb_2_Br_9_ and Cs_3_Bi_2_Br_9_ microcrystals, respectively).^33^ Therefore, they have been successfully used as photocatalysts in oxidation reactions, such as ring-opening and C(sp^3^)–H activation reactions.^29,33−35^ The photocatalytic oxidation of benzyl alcohols and toluene by Cs_3_Bi_2–*x*_Sb_*x*_Br_9_ NCs (*x* = 0–2) occurs via a C–H activation mechanism involving the photogenerated holes (h^+^), which originate in the VB of the photocatalyst, and oxygen radical anions (O_2_^•–^) active species that are produced after electron transfer from the CB to oxygen molecules.^33,36^ Moreover, the presence of a small amount of Sb cations in the host lattice of Cs_3_Bi_2_Br_9_ boosts the photocatalytic activity for the selective oxidation of toluene by enhancing the separation and transfer of photogenerated charges to the photocatalyst surface and also increases their lifetime.^25,37^ The photocatalytic properties of A_3_B_2_Br_9_ are primarily influenced by the nature of the B cation; however, the type of A cation also plays a key role. A-site cations can cause the distortion of [SbBr_6_]^3–^ octahedra in the metal halide structure due to the Jahn–Teller effect and, consequently, affect the crystal field splitting energy of the ions at B-sites, thereby varying the photocatalytic activity of the metal halide. In particular, A_3_B_2_Br_9_ NCs prepared with Cs^+^ exhibit a higher photocatalytic activity for the oxidation of toluene in comparison with those based on CH_3_NH_3_^+^.^38^

Yet, in terms of photocatalytic reduction reactions, Cs_3_Sb_2_Br_9_ NCs have been tested only for the CO_2_ reduction to CO.^28,39^ Hereby we explored the suitability of lead-free Cs_3_Sb_2_Br_9_ nanocrystals for photocatalytic reduction transformations of benzyl bromides and demonstrate the potential of lead-free Cs_3_Sb_2_Br_9_ NCs to photocatalyze the reduction of *p*-substituted benzyl bromides (specifically, *p*-^t^BuC_6_H_4_CH_2_Br, *p*-CH_3_OC_6_H_4_CH_2_Br, *p*-ClC_6_H_4_CH_2_Br, *p*-BrC_6_H_4_CH_2_Br, and *p*-NO_2_C_6_H_4_CH_2_Br) and produce C–C coupling products in the absence of a cocatalyst. Cs_3_Sb_2_Br_9_ NCs not only had a better photocatalytic activity (turnover number, TON) and photostability compared to those reported for CsPbBr_3_ perovskite NCs, but they could also be reused for up to 3 times. Furthermore, we could achieve a good control over the selectivity of the reaction by tuning the electron donor properties of the substituents.

In this work, Cs_3_Sb_2_Br_9_ NCs were synthesized by a hot injection strategy, following a reported protocol with some modifications.^40,41^ Briefly, Cs_2_CO_3_, Sb(CH_3_CO_2_)_3_, octadecene (ODE), oleic acid (OA), and oleylamine (OLA) were mixed in a three-necked flask, and the mixture was heated to 140 °C for 1 h under vacuum. Then, benzoyl bromide in ODE was swiftly added to the mixture under nitrogen, and the reaction was immediately quenched. The as-synthesized NCs were characterized by UV–visible spectroscopy, X-ray diffraction (XRD), electron microscopies, and thermogravimetry. The colloidal dispersion of Cs_3_Sb_2_Br_9_ NCs presented two excitonic bands at ca. 436 nm and 374 nm (Figure 1a) in agreement with previous results.^42^ The main crystalline planes in the XRD pattern were observed at 22.5° (012), 27.6° (201), 32.0° (022), 39.5° (212), and 45.9° (204), which correspond to the pure Cs_3_Sb_2_Br_9_ metal halide with a hexagonal crystalline phase (ICSD-39824, Figure 1b) and a 2D-layered structure with *P*3̅*m*1 space group (Figure 1c).^43^ Transmission electron microscopy (TEM) images show that the product is characteristic of heavily aggregated NCs (Figure 1d). The average size of the crystallites was estimated via the Scherrer equation (considering the XRD peaks at 31.99°; 39.45° and 45.87°; see the Supporting Information for the detailed analysis) which yielded a value of 18.3 nm.^44^ As a result of the rapid degradation of this material under the electron beam, low exposure in the scanning electron microscopy (SEM) measurement was used for the morphological analysis (according to which the nanoparticles were found to be roughly spherical; see Figure S1a,b). High-resolution transmission electron microscopy (HRTEM) further confirmed that the NCs exhibited a hexagonal 2D-layered structure (Figure S1c,d). The elemental analysis performed via SEM energy-dispersive X-ray spectroscopy (EDX) returned a Cs:Sb:Br ratio of 2.5:2:8, consistent with the expected Cs_3_Sb_2_Br_9_ stoichiometry (Figure S2a and Table S1). The elemental mapping from the SEM images clearly showed that Cs, Sb, and Br are homogeneously distributed in the sample (Figure S2b–e). Thermogravimetry analysis (TGA) indicated that 45.1% of weight loss was ascribed to the adsorbed toluene to the NCs surface as has been previously reported for other NCs,^45,46^ whereas 30.3% of the sample was composed by the organic ligands (both bound and free ligands) and the inorganic NC cores accounted for the remaining 24.6% (Figure S3)^47^

**Figure 1:**
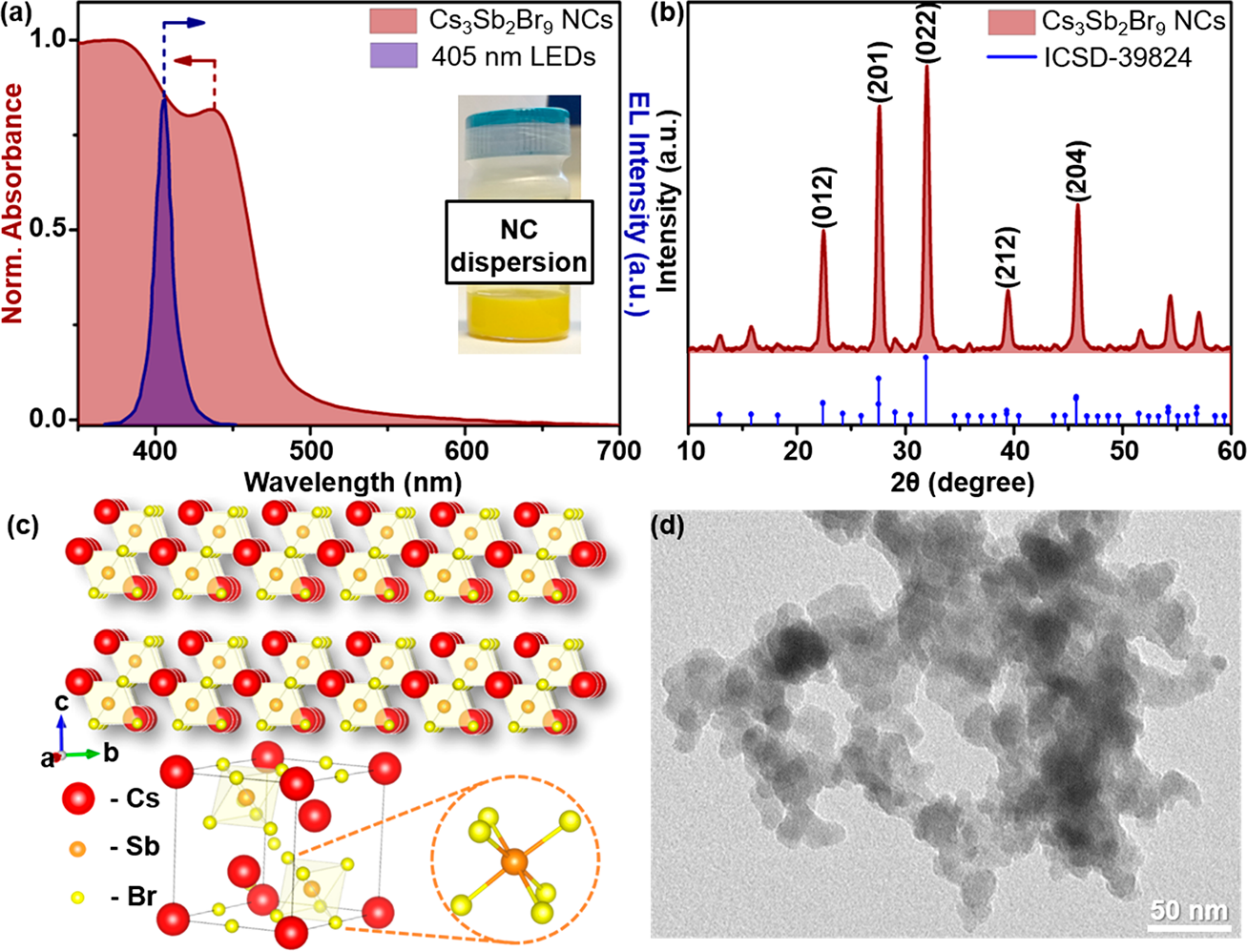
(a) Normalized absorbance of the Cs_3_Sb_2_Br_9_ NCs (in dark red) and overlap with the 405 nm LED emission (in violet) used as excitation source in the photocatalytic reactions; inset: nanocrystal dispersion; (b) XRD patterns of the corresponding NCs with the reflection of bulk material (ICSD number 39824); (c) crystal structure of Cs_3_Sb_2_Br_9_ NCs, its unit cell, and the SbBr_6_ octahedra; (d) representative TEM images of Cs_3_Sb_2_Br_9_ NCs. Scale bar: 50 nm.

The redox properties of the Cs_3_Sb_2_Br_9_ NCs were evaluated by combining optical and electrochemical measurements. Figure S4a depicts the Tauc plot from the absorption spectrum used for the calculation of the direct energy gap (Δ*E*^opt^ = 2.58 eV), which is close to the reported value of 2.45 eV,^37^ while Figure S4b reports the cyclic voltammetry plot with an irreversible cathodic peak potential for Cs_3_Sb_2_Br_9_ (−1.50 V vs Ag/AgCl reference electrode, where there is a higher density of states), attributed to the injection of electrons into the CB of the NCs. Considering the half-wave oxidation potential of the external Fc/Fc^+^ standard (0.52 V vs Ag/AgCl reference electrode), the CB energy level of the Cs_3_Sb_2_Br_9_ NCs was estimated as −2.78 eV vs vacuum (see the Supporting Information for further details, specifically the section on the electrochemical properties of Cs_3_Sb_2_Br_9_ NCs), which is close to the reported value of −2.98 eV for Cs_3_Sb_2_Br_9_ microcrystals.^33^ However, as the oxidation peak potential was not well resolved, the VB energy level (−5.36 eV) was calculated from the optical energy gap and the CB value (this is a common practice to determine the band gap energy levels of semiconductor materials).^48^

These data indicate that the electron transfer process from the Cs_3_Sb_2_Br_9_ NCs CB to the lowest unoccupied molecular orbital (LUMO) of the benzyl bromide substrates (−3.0 eV) should be thermodynamically favorable, with a driving force of −0.22 eV (see inset of Figure S4b on the reduction potentials of Cs_3_Sb_2_Br_9_ NCs alongside with the reduction potential of benzyl bromide), which is greater than the one observed for CsPbBr_3_ NCs.^24^ The redox properties of the systems, together with other factors, such as substrate interaction with the surface, the nature of the photocatalyst, the substrate preconcentration, and the colloidal stability (which will be discussed below), contribute to determining the photocatalytic properties of the Cs_3_Sb_2_Br_9_ NCs.

The photocatalytic activity of Cs_3_Sb_2_Br_9_ NCs was initially tested with benzyl bromide (**1a**) as the substrate (entry 1, Table 1) using an in-house developed high throughput screening photoreactor (Supporting Information, see general information photograph 1) that is able to set up 25 reactions with an LED emitting at 405 nm and temperature control at 30 °C; the tests used methanol (MeOH) as the sacrificial electron donor, N_2_ atmosphere, and irradiation in toluene for 48 h.^49^Tables 1 and S2 summarize the optimization of the photoreaction conditions, the standard conditions selected, and the control experiments in the photoreduction of benzyl bromide. As has been previously reported,^38^ photogenerated holes can oxidize toluene to benzyl radicals which can evolve to benzaldehyde in the presence of oxygen. A control experiment performed with **1a** under aerobic conditions showed that benzaldehyde and benzyl alcohol were the only products detected by GC-MS (Figure S5a). This reaction would be mediated by the superoxide anion radical (O_2_^•–^) generated by the reaction of oxygen with the photogenerated electrons. Moreover, in the absence of the substrate and under N_2_ atmosphere, bibenzyl product (**1b**) was obtained in only a 0.06% yield (entry 5), while no transformation was detected under O_2_ atmosphere (Figure S5b). These data indicate that the oxidation of toluene, acting as solvent and substrate in this case, by the photogenerated hole of Cs_3_Sb_2_Br_9_ NCs occurs with a very low efficiency as expected due to its oxidation potential of −6.7 eV.^50^ A similar behavior has been observed for the activation of C(sp^3^)–H bonds using Cs_3_Bi_2_Br_9_.^38,51^ Other control experiments performed in the absence of the NCs (entry 6) or light (entry 7), under otherwise the same conditions, resulted in the absence of products.^52^

**Table 1:**
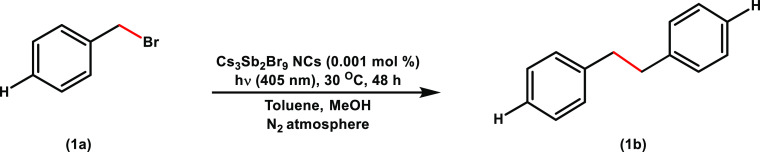
Standard Conditions and Control Experiments for Benzyl Bromide Reduction^a^

**Entry**^a^	**Variance**	**Solvent**	**Conversion (%)**	**1b (%)**
1	None	Toluene	100	77.0^b^
2	None	Hexane	100	33.7
3	None	Chlorobenzene	100	23.7
4	None	Ethyl Acetate	100	14.3
5	Without substrate	Toluene	–	0.06^c^
6	Without Cs_3_Sb_2_Br_9_ NCs	Toluene	n.d.^d^	n.d.
7	Without light	Toluene	n.d.	n.d.
8	Without MeOH	Toluene	28.9	16.0

a Conditions: Benzyl bromide (0.034 mmol), Cs_3_Sb_2_Br_9_ NCs (0.001 mol %), MeOH (0.68 mmol/20 equiv), in 2 mL of toluene after 48 h of irradiation (λ_max_= 405 nm) at 30 °C under N_2_. Yields determined by GC-MS (gas chromatography coupled with mass spectrometry) using biphenyl as internal standard.

b Homocoupling product exclusively produced from the substrate.

c Homocoupling product exclusively produced from the toluene oxidation

d n.d. = not detected.

Surprisingly, **1b** was obtained with a yield of 16% in the absence of methanol (entry 8), but with ca. 29% conversion, which suggests that the presence of the alcohol as the sacrificial electron donor is relevant for carrying out the reaction with a good chemical yield.

The polarity of the solvent can influence the conformation of the ligands bound to the NC, thereby enabling or hindering selective phototransformations by varying the degree of available space close to the NC surface.^53^ The kinetics of the catalytic process could be controlled by the diffusion coefficient of the substrate through the NC-ligand interphase and the solvation free energy, the latter being the result of both the ligand–substrate and the solvent–substrate interactions. Moreover, the solvent nature can have an influence on (i) the interaction between the substrate and the NC surface and (ii) the C–Br bond breaking to generate the benzyl radical and the bromide anion intermediates, and their corresponding adsorption onto the NC surface. In this study, the solvent nature had a critical role in the photoreduction of **1a**: benzyl bromide was fully converted when toluene was employed as the solvent, yielding the homocoupling product **1b** at 77%, while the use of the other solvents such as hexane, chlorobenzene, and ethyl acetate (entries 2–4, Table 1) lowered the selectivity toward **1b**.

The photocatalytic reaction was then evaluated for different *p*-substituted benzyl bromides using toluene and hexane as solvents (Table 2), and the products were analyzed by gas chromatography (GC) coupled with mass spectrometry (MS; see also the product characterization section in the Supporting Information, Figures S6–S16, and Table S3). When carrying out the reaction in toluene, the following products were observed: (i) the Csp^3^–Csp^3^ homocoupling products **b** by coupling of benzylic radicals coming from substrate **a**; (ii) Csp^3^–Csp^3^ heterocoupling products **c**, coming from the coupling of the benzylic radical from toluene and that of substrate **a**; and (iii) the products **d** obtained in the dehalogenation of substrate **a**. It is noteworthy that benzyl radicals coming from toluene can lead to the formation of bibenzyl product with a very low chemical yield (0.006−0.026% relative to the initial amount of toluene; see Table S4), suggesting that the oxidation of toluene by the hole occurs with a very low efficiency as was commented above. However, in the case of hexane, only the Csp^3^–Csp^3^ coupling and dehalogenation products were observed, allowing the analysis of the substituent effect in this solvent. Figure 2 summarizes the proposed mechanism for the photoreductive dissociation of benzyl bromides by Cs_3_Sb_2_Br_9_ NCs in both solvents upon visible light excitation (405 nm). Concerning the oxidation pathway, the use of methanol as a hole scavenger is well-known and established for the synthesis of valuable organic compounds by means of heterogeneous photocatalysts^54−56^ and in studies of photoinduced electron transfer processes.^57,58^ The effect of methanol oxidation in the formation of the photoreductive C–C coupling products was analyzed. The incorporation of methoxy radical was only observed in the case of 4-methoxybenzyl bromide, where 4-methoxybenzyl methyl ether was generated in 16.3% (entry 7, Table 2). These data suggest that formaldehyde and hydrogen were the main oxidation products of methanol in these organic solvents.

**Table 2:**
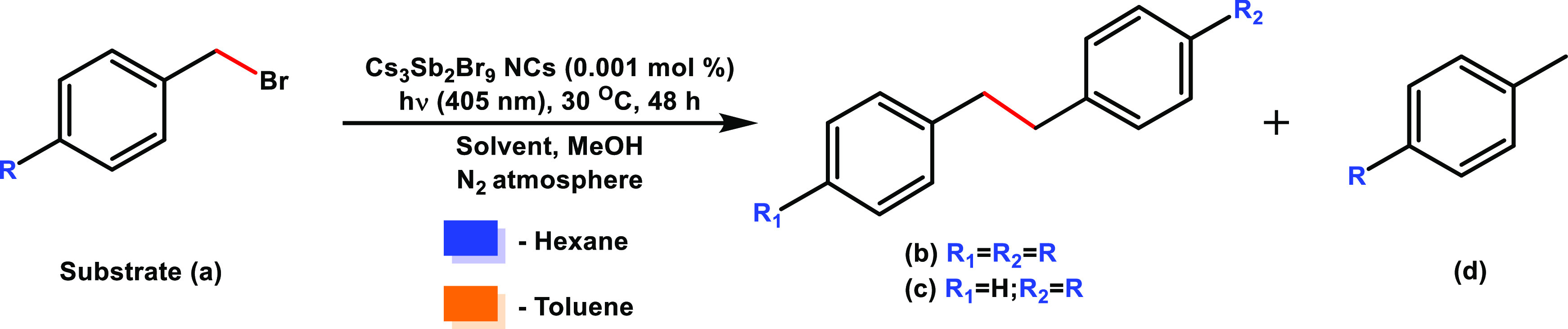

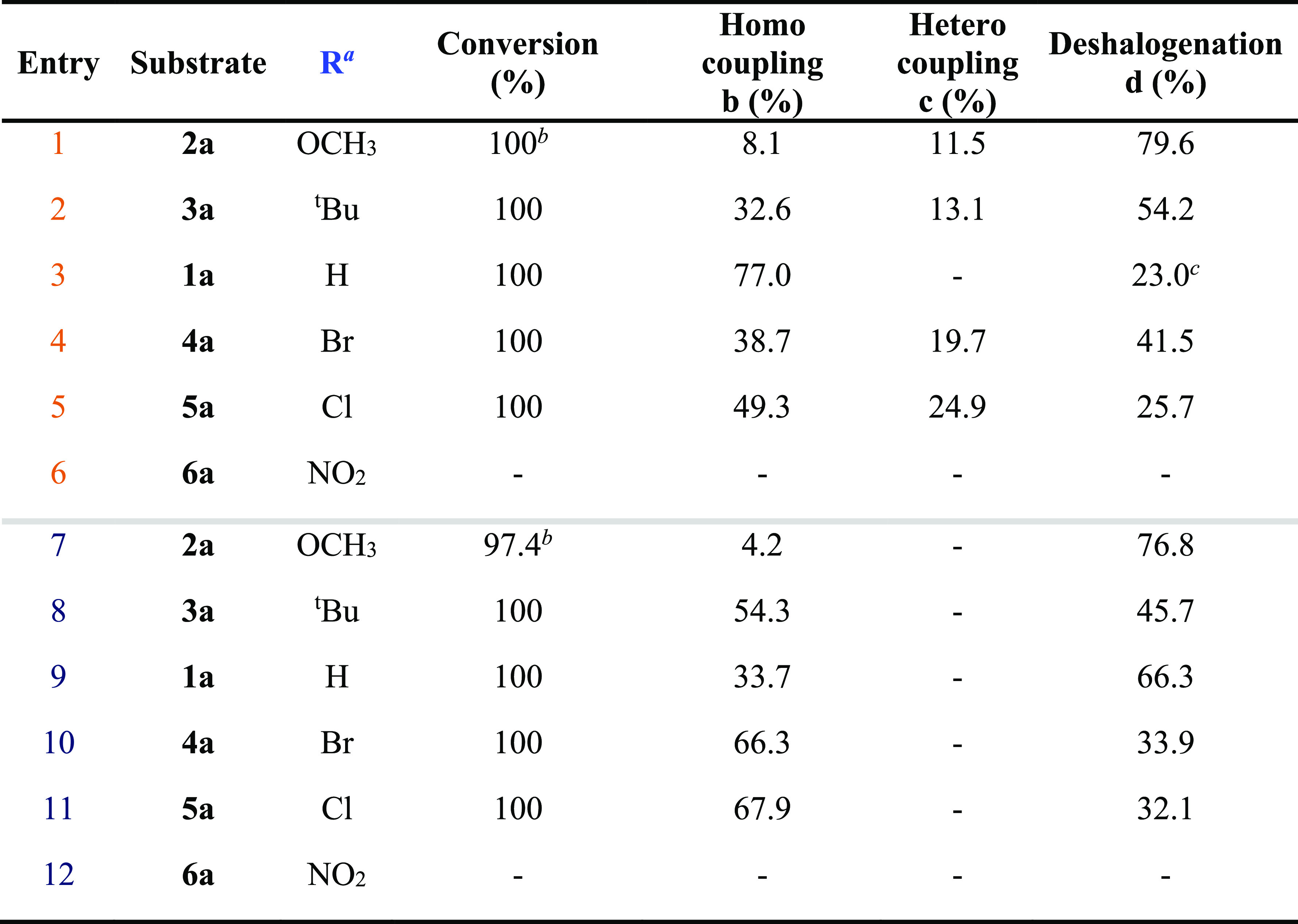
Reaction Scope for *p*-Substituted Benzyl Bromide Photoreduction

a Substrates (column 2) are organized depending on the *p*-substituent nature from electron-donating to electron-withdrawing groups (R = OCH_3_, ^t^Bu, H, Br, Cl, and NO_2_).

b In addition to the products indicated for *p*-methoxybenzyl bromide in entries 1 and 7, 4-methoxybenzyl methyl ether was obtained as a side product (16.3% in hexane and 1.1% in toluene). The formation of this side-product was indicative of the presence of methoxy radical cations in the reaction media, produced after the consumption of MeOH as electron donor.

c Yield calculated by subtracting the yield of the coupling product **1b** to the full conversion of substrate **1a**. No other products were observed. Average from duplicate measurements.

**Figure 2:**
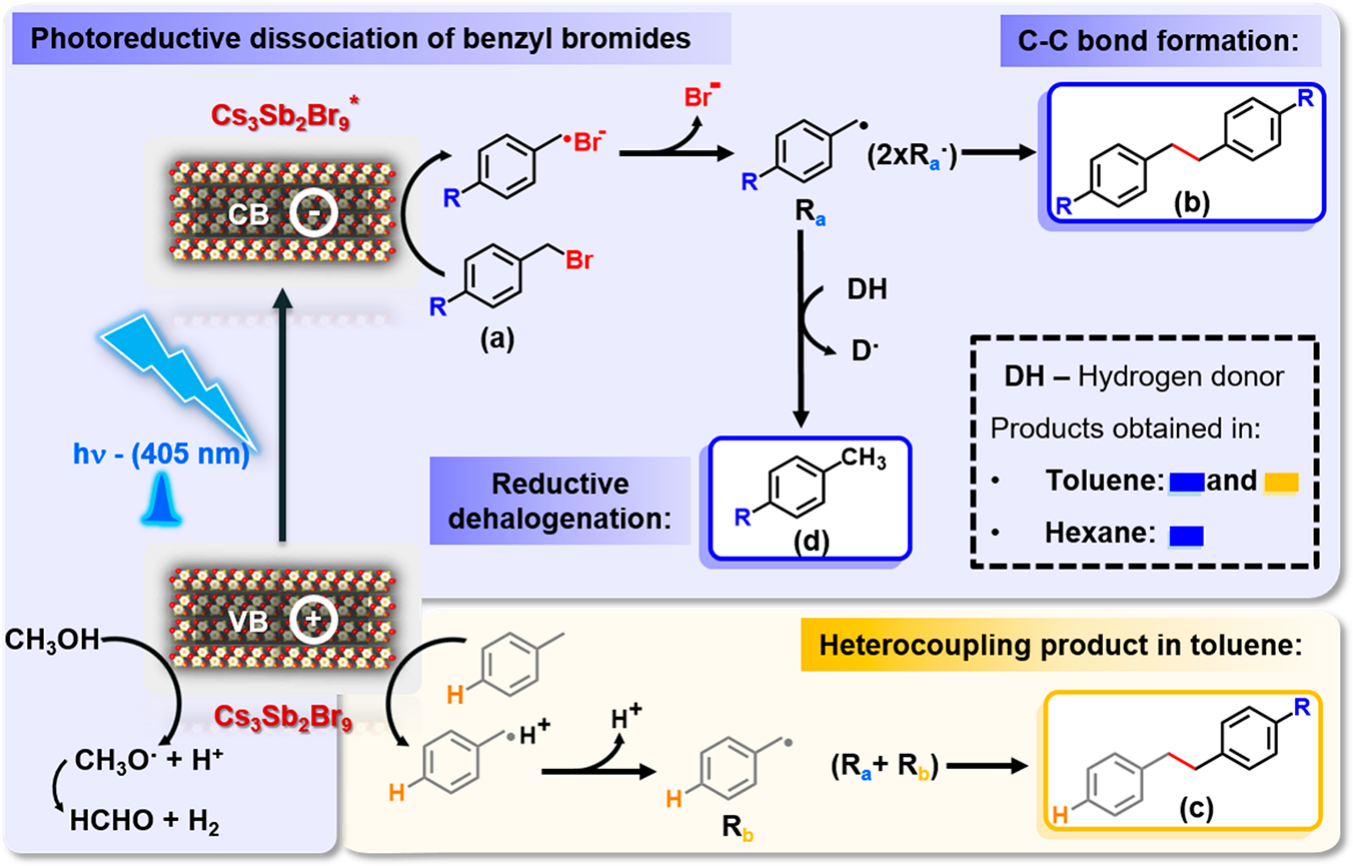
Scheme of the proposed mechanism for the photoreductive dissociation of benzyl bromides (**a**) conducted by Cs_3_Sb_2_Br_9_ NCs in hexane and toluene. The blue area represents the photoreductive dissociation of benzyl bromides to give rise to the C–C coupling (**b**) and dehalogenated products (**d**), whereas the orange area provides information about the additional heterocoupling product (**c**) observed in toluene. R_a_ and R_b_ are the benzyl radicals coming from substrate reduction and toluene oxidation, respectively.

After the exciton formation, the electron is transferred to the substrate, generating a radical anion, thus eventually leading to a bromine anion and a benzyl radical. The number and yield of products can be modulated (Table 2) via the type of solvent and the electron-donating/electron-withdrawing nature of the R substituent. Interestingly, a good correlation (*R*^2^ = 0.99) between the percentage of C–C homocoupling product and the sigma value (*σ*_*p*_) for the OCH_3_ electron-donating group, *t*-butyl electron releasing group, and electron-withdrawing groups (Cl and Br) in the *p-*position of the aromatic ring was obtained in hexane (Figure S17a).^59^ The following trend for the C–C homocoupling product formation was observed: OCH_3_ < H < ^t^Bu < Br ≈ Cl (compare data in Table 2). It is noteworthy that the ^t^Bu bulky moiety induced a higher degree of C–C homocoupling reaction probably due to a weaker interaction of the benzyl radicals from this substrate with the NC surface due to steric effects.^60^Figure S17b shows the correlation between the percentage of the dehalogenation product and the sigma value (*σ*_*p*_), which is favored by the electron-donating substituent. In the case of *p*-nitrobenzyl bromide, the photoreduction did not work in any solvent, which could indicate a different interaction of the substrate with the NC surface that avoids the formation of the C–C homocoupling.

According to data in Table 2, the selectivity in the C–C homocoupling product formation was mainly dependent on the solvent nature for substrate **1a** (runs 3 and 9). However, the electronic effects governed the product formation in the case of the *p*-substituted benzyl bromides **2a**–**5a**, obtaining a higher C–C coupling yield for the substrates with electron-withdrawing groups (Cl, Br). The selectivity observed for the C–C homocoupling product seems to be related to (i) the degree of the substrate–Sb^3+^ interaction on the NC surface and (ii) the adsorption of the benzylic radical (benzylic hydrogen atoms present a positive charge distribution) to the NC surface.^30,33^

The interaction of substrate **1a** with the surface atoms of the metal-free NCs was analyzed by Fourier transform infrared (FTIR) and nuclear magnetic resonance (NMR) spectroscopy. The mixture of Cs_3_Sb_2_Br_9_ NCs and **1a** was studied by ^1^H and ^13^C NMR spectroscopy using toluene-d8 as solvent. The methylene protons (Ha, Figure 3a) and the benzylic carbon (Figure 3b) shifted to higher field by Δppm = −0.03 and −0.12, respectively. This observation suggests that the bromide electronegativity was modified due to its interaction with the NC surface cations.^61^ Furthermore, the FTIR spectra of this mixture was compared to the spectra of the substrate, organic ligands, and the pristine NCs (Figure 3c). The C–Br stretching band of the substrate (at ca. 600 cm^–1^) underwent a shift to higher wavenumbers; specifically, Δν = 4 cm^–1^. Similar trends were observed for *p*-methoxybenzyl bromide (**2a**; Figure S18) and *p*-chlorobenzyl bromide (**5a**; Figure S19) with a Δν = 8 and 13 cm^–1^, respectively. All these data confirm the adsorption of the substrate to the NC surface. Similar shifts toward higher energy have been previously reported for the interaction of anchoring groups of functional organic molecules close to the surface of perovskites NCs, such as the C=O of terephthalic acid^62^ or the N–H of benzylamine.^63^

**Figure 3:**
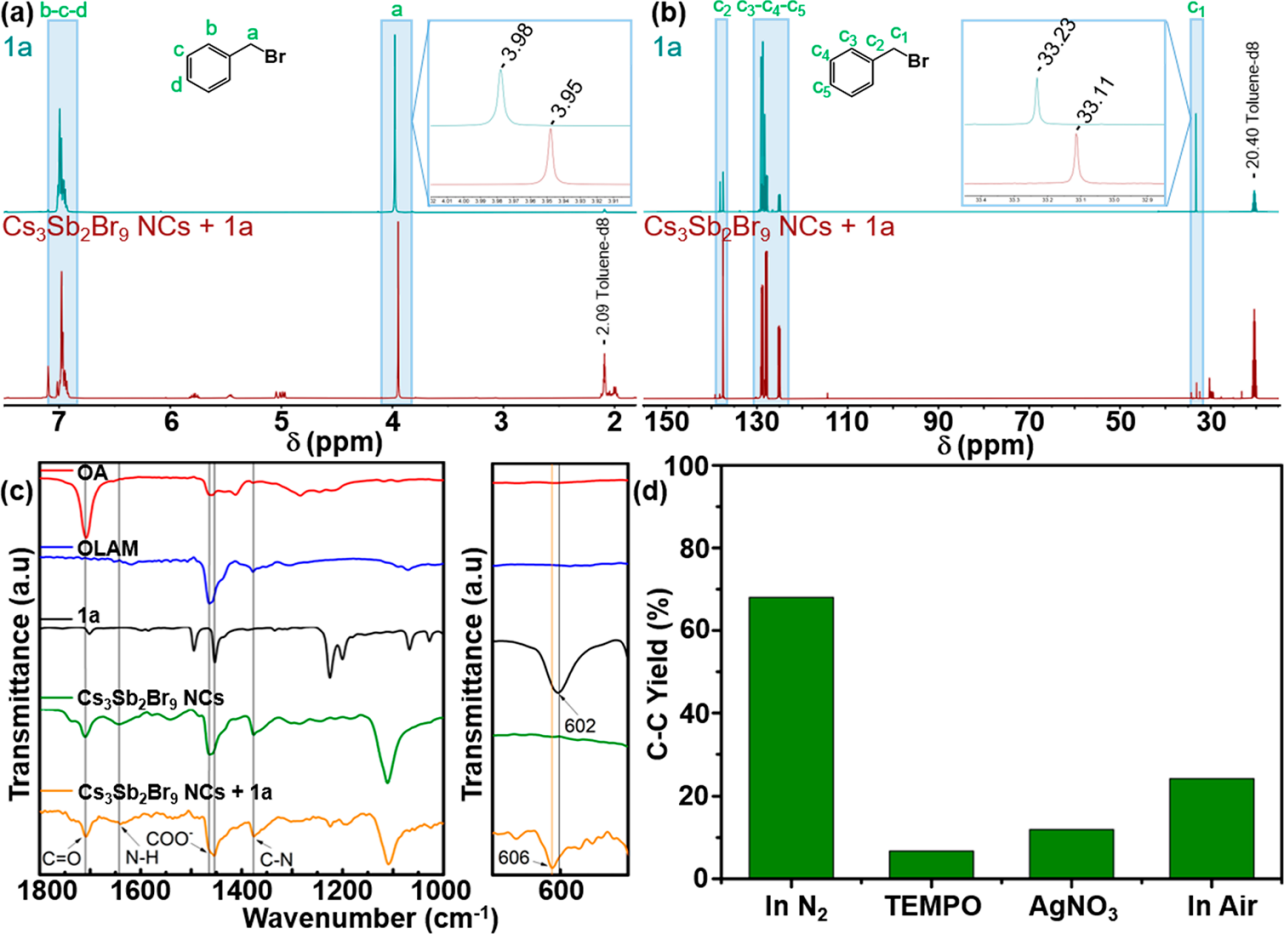
(a) ^1^H NMR and (b) ^13^C NMR of a **1a** stock solution (blue line) and Cs_3_Sb_2_Br_9_ NCs plus **1a** (red line) in toluene-d8. (c) FTIR spectra of OA (red line), OLAM (blue line), **1a** (black line), Cs_3_Sb_2_Br_9_ NCs (green line), and Cs_3_Sb_2_Br_9_ NCs plus **1a** (orange line) and FTIR zoom of the C–Br stretch region. (d) Photocatalytic activity of Cs_3_Sb_2_Br_9_ NCs in the absence or presence of various active species scavengers, such as TEMPO, AgNO_3_, and air, for the C–C coupling of **5a**.

To provide evidence on the radical mechanism of the photoreduction of benzyl bromide substrates by Cs_3_Sb_2_Br_9_ NCs, the photocatalytic reaction was performed in the presence of the radical scavenger 2,2′,6,6′-tetramethylpiperidine-1-oxyl (TEMPO) as well as two electron acceptors, namely, AgNO_3_ and molecular oxygen. The *p*-Cl-benzyl bromide (**5a**) was used as substrate because it led to a higher yield of bibenzyl products in hexane (see experimental details in the Supporting Information). The C–C coupling product formation was drastically reduced from 69% to 6%, 10%, and 25% for TEMPO, AgNO_3_, and in air, respectively (Figure 3d). These results agree with the radical trapping intermediates by TEMPO and the competition of AgNO_3_ and O_2_ (air atmosphere) to accept the photogenerated electron of the NC, thereby reducing Ag^+^ and forming the superoxide anion radical (O_2_^•–^) species, respectively. Interestingly, the formation of the 4-chlorobenzyl radical-TEMPO adduct was observed by GC-MS (Figure S20), which certainly confirmed that the mechanism involves the formation of the benzyl radical (Figure 2).

Considering the electronic properties of the substrate in the photoreduction of *p*-substituted benzyl bromides in the presence of Cs_3_Sb_2_Br_9_ NCs, the ability of the intermediate species formed to bind the NC surface, and the experimental evidence noted above, the following mechanism can be proposed (Figure 4): (i) adsorption of the substrate to the NC surface facilitated by the Sb–substrate interaction and photogeneration of the electron (e^–^) and hole (h^+^) under visible light excitation of Cs_3_Sb_2_Br_9_ NCs (step 1) as was evidenced by FTIR and NMR spectroscopy; (ii) charge transfer to generate the methoxy radical (MeO^•^, that eventually produce formaldehayde and hydrogen, h^+^ transfer) and benzyl bromide radical anion (ArCH_2_Br^•–^, e^–^ transfer; step 2); (iii) dissociation of the radical anion forming the benzyl radical (ArCH_2_^•^ which was trapped by TEMPO forming the adduct ArCH_2_–TEMPO) and the bromide anion (Br^–^ that can form Br_2_ or HBr), followed by their adsorption to the NC surface ions (onto Br and Sb surface sites, respectively, step 3) ; (iv) product formation (step 4). A weak adsorption of the benzyl radicals to the NC surface would result in the formation of C–C coupling products, while a strong adsorption of the benzyl radicals to the NC surface would give rise to the dehalogenation product.^64^ Therefore, following our hypothetical model, Sb would play a triple role in this photocatalytic process since it (i) facilitates the adsorption of the substrate to the NC surface, (ii) mediates the electron transfer, and (iii) interacts with the bromide anion formed after the charge transfer.

**Figure 4:**
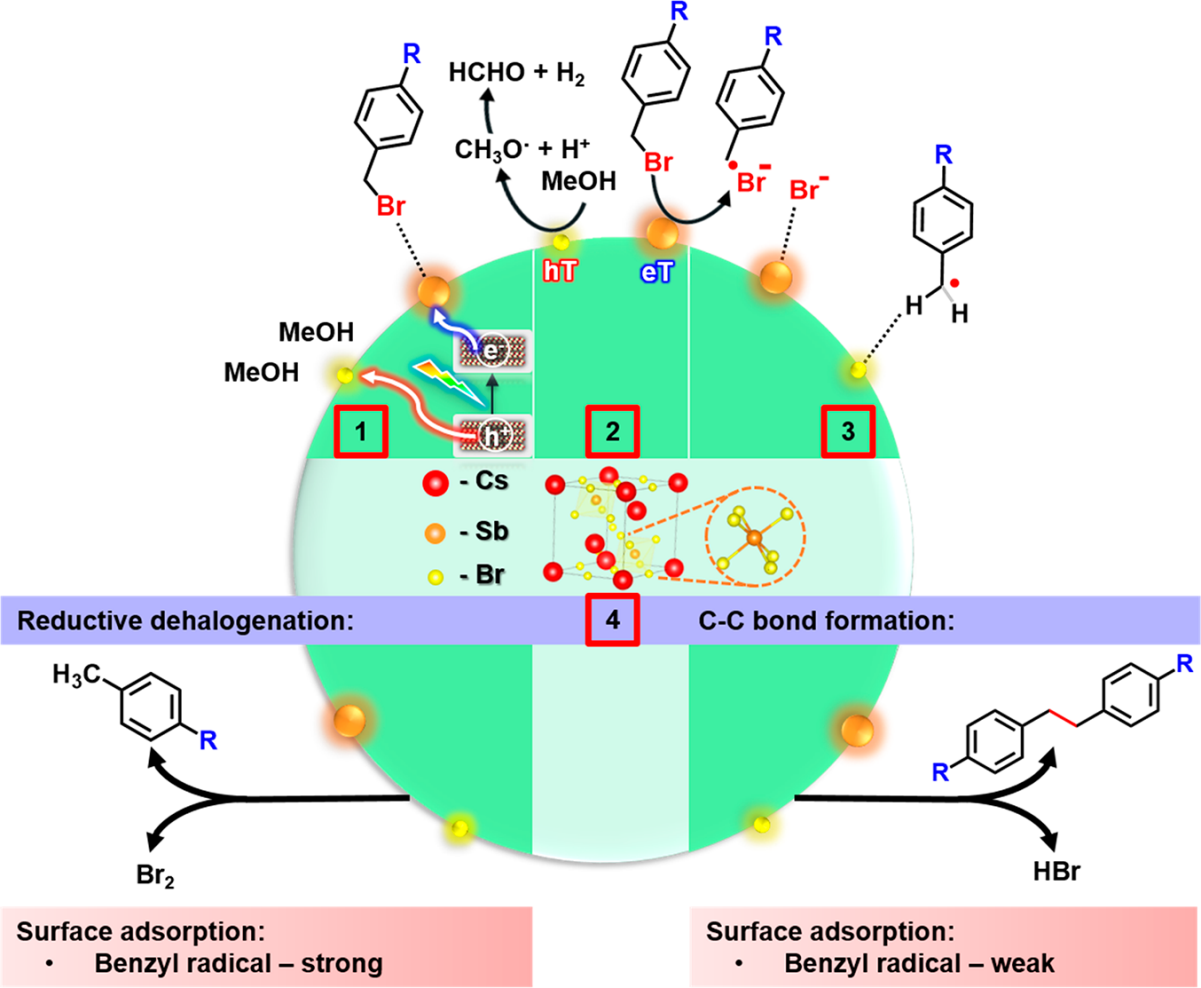
Proposed mechanism of the photocatalytic benzyl bromide photoreduction: (1) photogeneration of the electron and hole, charge diffusion, and substrate-surface approximation; (2) electron transfer to the substrate to generate the radical anion of the benzyl bromide and hole transfer to the MeOH (formaldehyde and H_2_ formation); (3) adsorption of the Br anion and benzyl bromide radical to the NC surface; the strength of the binding of the benzyl radical to the NC surface would depend on the electronegativity of the substituent at the *p*-position; and (4) competitive formation of the reductive dehalogenation product vs the C–C coupling product.

On this matter, the C–H activation of toluene by Cs_3_Sb_2_Br_9_ has been studied using density function theory (DFT) analysis by Shi et al.^33^ It was proposed that methylbenzene adsorbs on the Br sites by attractive Columbic interactions and the calculated bond lengths between the hydrogens of the benzyl radical and the Br sites of the NCs were shorter after the C–H activation, thus suggesting that the radicals were closer to the surface. This observation also agrees with the interaction between the benzylic hydrogen with Br surface atoms described by Zhang et.al.^38^ It is also expected that the Br^–^ anion interacts with the cation at the NCs surface, preferentially the Sb^3+^ cations given their higher positive charge density in comparison with Cs^+^.

As a control experiment, the photoreaction was performed in the absence of the substrate to evaluate the origin of the Br in molecular Br_2_. The supernatant obtained after the centrifugation step (Figure S21) was colorless, and no absorption was detected, thereby indicating that the charge transfer-complex between Br_2_ and aromatic compounds was not formed (further explanation below), thus ruling out the possible Br^–^ anion leaching from the lead-free metal halide surface. Hence, after the photoreduction of substrates **2a**, **3a**, **4a**, and **5a**, the crude reaction mixtures were centrifuged and the supernatants were analyzed by absorption spectroscopy (Figure S22) in order to detect the formation of molecular Br_2_, expected as a product in the reductive dehalogenation (Figure 4). The brownish color of the samples was indicative of the formation of molecular Br_2_ and became more intense by increasing the electron donor character of the benzyl substitutent (photographs in Figure S22). However, a direct measurement of molecular Br_2_ was not possible since it forms charge transfer complexes with aromatic compounds, as was previously reported for Br_2_–benzene complexes (absorption band ca. 300 nm).^65−67^ These complexes featured a well-defined UV band at 350 and 335 nm for **2a** and **3a**, respectively, while a shoulder at 330 nm was observed for **4a** and **5a**. Moreover, two additional experiments were carried out to confirm the generation of molecular Br_2_ in the reaction media (see further information in SI section 3.6, Figure S23), namely, addition of KOH to the reaction mixture or direct irradiation of the mixture at 365 nm broke down the complex and produced the photoaddition of Br_2_ to the aromatic compound.

The performance of the photocatalyst was analyzed after one photocatalytic cycle by different techniques. The XRD patterns of the Cs_3_Sb_2_Br_9_ NCs before and after the photocatalytic cycle indicated that the crystalline structure of the NCs was preserved (see Figure S24). Concerning the optical properties, the absorption spectrum of the Cs_3_Sb_2_Br_9_ NCs remained almost unchanged after the reaction, although a small red-shift (Figure S25a) and an increase in the light scattering were observed. This variation could be ascribed to the partial removal of ligands and, as a consequence, to a slight colloidal NC aggregation after the centrifugation step. However, no relevant changes in the NC morphology were observed (Figure S25b,c). From the XPS analysis (Figure S26), we can conclude that the main signals of Cs_3_Sb_2_Br_9_ NCs (Cs_3d_, Sb_3d_, and Br_3d_ peaks) appeared at similar positions in the three samples, before and after the photocatalytic cycle carried out in toluene and hexane for *p*-bromobenzyl bromide. In the case of the sample in hexane, a new Br_3d_ doublet with the main peak at 70.5 eV was observed; a comparison with literature suggests that this new Br species could be related to C–Br bonds, as has been described in brominated tetraphenyl porphyrins and graphite bromide.^68,69^ Therefore, this peak was assigned to the C–Br bonds of 4,4′-dibromobibenzyl, coming from the bromide C–C homocoupling, which could slightly precipitate during the purification and product isolation steps due to its low solubility in hexane.

Finally, the reusability of the photocatalyst was assessed. After the first photocatalytic cycle, the NCs were precipitated from the reaction solution via centrifugation. Then, the precipitate was collected and reused in the next cycle. Although the amount of the collected photocatalyst was smaller than the starting one, its photocatalytic activity was maintained for up to three cycles, with a standard deviation between the different cycles of 5% (Figure S27a; see the Supporting Information for further details). The absorption spectrum of the NCs after being used in three cycles of photoreduction conducted in the presence of MeOH (sacrificial electron donor) evidenced a broadening of the excitonic peak together with a 10 nm red shift and an intensification of the scattered light (Figure S27b). These signs as well as the comparison with the absorption spectrum of the NCs subjected to 3 photocatalytic cycles without MeOH (Figure S27c) could indicate that the polarity of this solvent reduces the colloidal stability of the nanomaterial (most likely because of a loss of ligands), thus leading to a partial NC aggregation, while the crystalline structure was preserved.

In summary, we have reported the first example of Csp^3^–Csp^3^ homocoupling of benzyl halides to bibenzyl derivatives promoted by a stable, efficient, and sustainable nanophotocatalyst, namely, lead-free Cs_3_Sb_2_Br_9_ NCs. Remarkably, the reaction was carried out under visible light irradiation and in the absence of a cocatalyst. The electron-donating/electron-withdrawing character of the benzyl bromide substituent at the *p*-position and the solvent type were found to determine the selectivity of the transformation producing higher homocoupling yields with electron-withdrawing groups, in both solvents. The photocatalyst preserved its performance after three photocatalytic cycles and provided C–C coupling products with a good chemical yield. This work opens up an avenue for the potential application of lead-free metal halides in photocatalysis to be applied in more complex organic syntheses.

## Supporting Information Available

NC synthesis, photocatalytic procedures, and characterization methods; calculation of the NC concentration, TON, and crystallite size; characterization of the photocatalyst by TEM, HR-TEM, EDS, TGA, and CV; optimization of the photocatalytic reactions and product identification by GC-MS; Br_2_ detection experiments; photocatalyst recyclability experiments by XRD, absorption, TEM, and XPS (PDF)
