# Supplementary material for: The Dark Side of Lead-Free Metal Halide Nanocrystals: Substituent-Modulated Photocatalytic Activity in Benzyl Bromide Reduction
Source: ACS Energy Lett. 2023 May 30;8(6):2789–98. doi: 10.1021/acsenergylett.3c00771 (PMC10262690; doi:10.1021/acsenergylett.3c00771)
Supplement: Supplementary file 1 — nz3c00771_si_001.pdf [file nz3c00771_si_001.pdf]

Supporting information

The dark side of lead-free metal halide nanocrystals: substituent-modulated photocatalytic activity in benzyl bromides reduction

*Ignacio Rosa-Pardo[1], Dongxu Zhu[2], Alejandro Cortés-Villena[1], Mirko Prato[3], Luca De Trizio[2], Liberato Manna[*2], Raquel E. Galian[*1] and Julia Pérez-Prieto[*1]*

1. Institute of Molecular Science, University of Valencia c/ Cat. José Beltrán 2, Paterna, 46980, Valencia, Spain.

2. Nanochemistry, Istituto Italiano di Tecnologia, Via Morego 30, 16163, Genova, Italy.

3. Materials Characterization Facility, Istituto Italiano di Tecnologia, Via Morego 30, 16163, Genova, Italy.

Table of contents

1. **General information** 2
2. **Supporting tables** 9
3. **Supporting figures** 11
   - 3.1. **Photocatalyst characterization** 11
   - 3.2 **Control experiments of the toluene oxidation** 14
   - 3.3 **Products characterization** 15
   - 3.5. **Mechanistic studies of the photocatalytic process** 21
   - 3.6. **Br_2 detection experiments** 23
   - 3.7. **Photocatalyst performance after photocatalytic cycles** 26
4. **References** 30

1. General information

Chemicals. Cesium carbonate (Cs_2CO_3, 99.9%), antimony acetate ($Sb(CH_3CO_2)_3$, 99.99%), 1-octadecene (ODE, 90%), oleylamine (OLA, 70%), oleic acid (OA, 90%), benzoyl bromide (Bz-Br, 97%), benzyl bromide (98%), *p*-tert-Butylbenzyl bromide (97%), *p*-Methoxybenzyl bromide (98%), *p*-Bromobenzyl bromide (98%), *p*-Chlorobenzyl bromide (98%), *p*-Nitrobenzyl bromide (97%), toluene (anhydrous, 99.8%), ethyl acetate (anhydrous, 99.8%), and hexane (anhydrous, 95.0%) were purchased from Sigma-Aldrich. Biphenyl (99.5%; GC grade) was purchased from TCI and methanol (anhydrous, 99.9%) was purchased from thermoscientific. *p*-Methoxybenzyl bromide was purchased from Apollo Scientific. All chemicals were used without any further purification.

Synthesis of $Cs_3Sb_2Br_9$ NCs.

Colloidal $Cs_3Sb_2Br_9$ NCs, were synthesized by hot-injection approach. In a typical synthesis, Cs_2CO_3 (0.25 mmol; 81.45 mg), $Sb(CH_3CO_2)_3$ (0.331 mmol; 99.06 mg), 4 mL of ODE, 0.6 mL of OLA, and 1.2 mL of OA were mixed in a 50 mL 3-necked round-bottom flask inside a nitrogen glovebox and then, the mixture was heated up to 140 °C under vacuum for 1 h. Afterward, a benzoyl bromide dispersion (210 µL/0.5 mL) in degassed ODE was swiftly injected inside the flask under N_2 atmosphere. Then, the reaction was immediately quenched in an ice−water bath and 4 mL of ethyl acetate was added to the crude NCs solution and centrifuged at 5500 rpm for 5 min. The final precipitate was dispersed in toluene (4 mL) and stored in a glove box for further use. All the washing procedures were carried out under an inert atmosphere.

General procedure for the photocatalytic reduction of benzyl bromides.

The photocatalytic reactions were prepared in a nitrogen glovebox and conducted in 10 mL gas-tight crimped vials under stirring (160 rpm) using an orbital shaker in a photoreactor with blue LEDs (405 nm) for 48 h at 30 °C. To prepare the reactions, 450 µl of a stock solution of the colloidal $Cs_3Sb_2Br_9$ NC dispersion was added to a vial, dried over the vacuum, and weighted for every single reaction (10.0 ± 0.5 mg). Then, inside the glove box, the substrate (100 µl of the *p*-substituted benzyl bromide stock solution, final concentration 17 mM), the electron donor (MeOH, 20 equivalents), and the corresponding solvent (V_F=2 mL) were added. Immediately after the reaction finished, the crude of the reaction was centrifuged at 12500 rpm for 15 minutes, and then, biphenyl (75mM) was added as the internal standard (IS) to the supernatant and subjected to GC-MS analysis to determine the conversion of substrates and the yield of the desired product. Both substrates and biphenyl were dissolved in ethyl acetate for those reactions performed in hexane, due to their low solubility in this solvent.

The reported yield of the product obtained was an average of at least 2-5 runs. The pellet was redispersed in toluene to recover the colloidal perovskite after the photocatalytic cycle.

Photocatalytic system.

Light source: The reactions were performed using (λ= 405±10 nm) LUXEON LED, mounted on a 10mm Square Saber - 1030 mW@700mA as a light source.

Temperature Control: Reaction temperature was controlled by a high-precision thermoregulation Hubber K6 cryostat. Likewise, to guarantee stable irradiation the temperature of the LEDs was set up at 21 °C.

The reactions have been carried out in an in-house parallel High Throughput Screening (HTS) photoreactor with a capacity to set up to 25 reactions with different excitation wavelengths, respectively, under high-intensity irradiation. These unique HTS platforms allow for tight control of the light intensity and the temperature of the reactions. The 25-positions photoreactor is operable at 1-15 mL reaction volumes for each reaction.

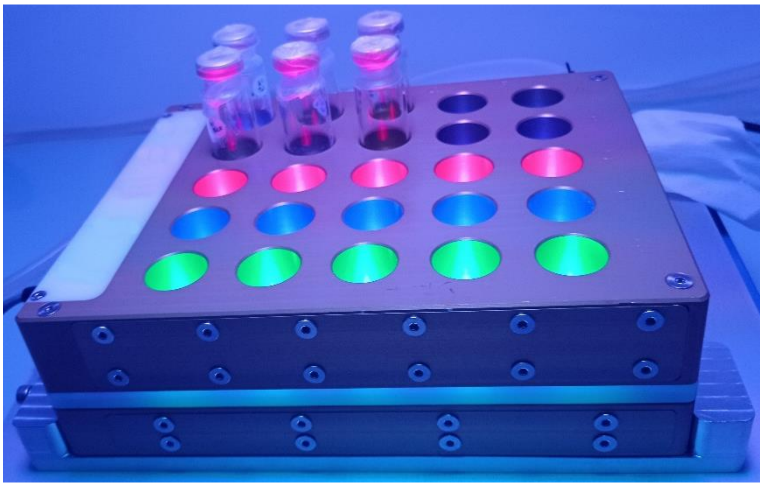

Photograph 1. In-house developed 25-well parallel photoreactor used for the reactions.

Optical Characterization.

The absorption spectra of the NCs dispersion were recorded using a Cary300 spectrophotometer and UV/VIS/NIR spectrophotometer Lambda 1050, equipped with software PerkinElmer UV Winlab-ink. The absorption spectra of the NCs in films were measured using a 150 mm InGaAs Integrating Spheres Module of Lambda 1050, equipped with software PerkinElmer UV Winlab-ink. The samples were prepared by diluting NCs samples in 2 mL of toluene in 1 cm path length quartz cuvettes with airtight caps. The NCs were prepared in films, by drop-casting 100 µL of NCs suspensions (5 mg/mL) over the center of a glass substrate (75x75 mm).

Thermogravimetric analysis (TGA).

Thermogravimetric analysis of the NCs was carried out with a Mettler Toledo TGA/SDTA851e/SF/1100 apparatus in the 25–800 °C temperature range under a 10 °C min^{-1} scan rate in a nitrogen atmosphere.

X-ray Diffraction (XRD) Characterization.

XRD patterns of the NCs were acquired with a Pananalytical Empyrean X-ray diffractometer equipped with a 1.8 kW Cu Kα ceramic X-ray tube and a PIXcel3D 2 × 2 area detector, operating at 45 kV and 40 mA. Specimens for XRD measurements were prepared by dropping a concentrated NC dispersion onto a silicon zero-diffraction single crystal substrate. The diffraction patterns were collected under ambient conditions using a parallel beam geometry and the symmetric reflection mode. After reactions, the XRD patterns were measured with a powder diffractometer Empyrean from Panalytical equipped with CuKα anode operated at 45 kV and 40 mA. Single scans were acquired in the 2θ=5° to 60° range with a step size of 2θ=0.01° in Bragg–Brentano geometry in air. XRD data analysis was conducted using the HighScore 4.1 software from Panalytical.

X-ray Photoelectron Spectroscopy (XPS) Characterization.

XPS spectra were collected on the sample before and after the photoreduction of *p*-Br-benzyl bromide 4a performed in toluene or in hexane. Few microliters of the samples' solutions were drop cast of clean Au substrates.

XPS data were acquired using a Kratos Axis UltraDLD spectrometer, using a monochromatic Al Kα source, operated at 20 mA and 15 kV. High resolution XPS scans, reported in figure S26, were acquired at pass energy of 20 eV, with an energy step of 0.1 eV, over an area of 300 x 700 microns.

The Kratos charge neutralization system was used during data acquisition. The binding energy scale was calibrated by setting the main line of the carbon 1s spectrum to 284.8 eV. Spectra were analysed using CasaXPS software (version 2.3.24).

Attenuated total reflectance-Fourier transform infrared spectroscopy

To ascertain the substrate approach to the $Cs_3Sb_2Br_9$ NC surface, the NCs and the substrates were mixed at the same concentration used in the photocatalytic reactions and the mixture was stirred for several hours in an orbital shaker to ensure the interaction between them. Then, the ATR-FTIR analyses were performed in a Bruker alpha II spectrometer by drop-casting 20 µL (2x10 µL) of the colloidal mixtures.

Nuclear magnetic resonance spectroscopy

The interaction of benzyl bromide with the surface of $Cs_3Sb_2Br_9$ NCs was also monitored by ^{1}H- and ^{13}C-NMR. A colloidal dispersion of the NCs and the substrate was prepared in toluene-d8 at the same ratio as in the photocatalytic reactions. Then, the colloidal dispersion was stirred in an orbital shaker for several hours and measured in a Bruker AV400 (400 MHz).

Gas chromatography–mass spectrometry

The work-up of the reaction was performed by adding the biphenyl (IS) to the crude. Then, 0.1 mL of the mixture was diluted with 0.9 mL of anhydrous toluene or hexane. The solution was injected into the GC and the products were identified according to the retention time and mass GC-MS (Agilent 7890B - 5977A).

A calibration curve was carried out using different concentrations of analytes (C_{AN}) in comparison to a given concentration of the internal standard (C_{IS}=75 mM). Then, it was analyzed the GC signals to compare the areas between analytes and internal standard generating a plot with a precise correlation. The coefficient between the area of the products and the IS used, allows us to quantify the product concentration of the reactions. This plot was used to study the products obtained.

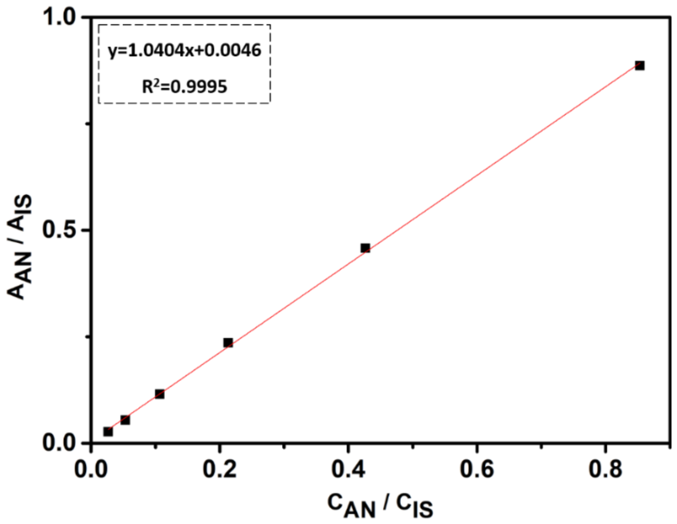

Correlation between the GC areas of the analytes / internal standard and their concentration.

The following equations were used to calculate the product yield (1) and substrate conversion (%; 2):

$$\boldsymbol{Product\ Yield}\ (\%) = \frac{[\boldsymbol{Product(AN)}]}{[\boldsymbol{Initial\ substrate}]} \boldsymbol{x100\%}\ \textbf{(1)}$$

$$\boldsymbol{Conversion}\ (\%) = \ \boldsymbol{100} - \frac{[\boldsymbol{Final\ substrate}]}{[\boldsymbol{Initial\ substrate}]} \boldsymbol{x100\%}\ \textbf{(2)}$$

Calculation of the photocatalyst active amount (mol %), turnover number (TON) and turnover frequency (TOF)

To calculate the amount (in mol %) of $Cs_3Sb_2Br_9$ NCs used for the photoreactions, it was followed the method used by Angshuman Nag et al.[1] for calculating the mol of $CsPbBr_3$ NCs and the molar extinction of this material. Firstly, a TGA of the NC dispersion was done in order to obtain the inorganic part of the photocatalytic system ($Cs_3Sb_2Br_9$ NCs + organic ligands + ODE). Thus, if it was weighted 10 mg of the photocatalytic system dried for every single reaction, only 2.5 mg (25 % of the material) corresponded to photoactive $Cs_3Sb_2Br_9$ NCs. Then, considering the round shape of a single NC and its diameter (18.3 nm), calculated by the Scherrer equation, was determined the weight of a single NC using the formula (1), which takes into account the density of the material (3.97 g/cm^3)[2, 3] and the volume of a nanosphere [$v=(4/3)\cdot\pi\cdot r^3$)]. The number of NCs in the photoreaction batch was determined by dividing the inorganic part of the NCs weighted by the weight of a single NC (2) and for the mol of NCs (3), was divided the number of NCs by Avogadro's number (N_A). Finally, the mol % of photocatalyst was obtained using the formula (4).

(1)

$$\boldsymbol{m_{NC}} = \boldsymbol{density}\ \left(\frac{\boldsymbol{g}}{\boldsymbol{cm^3}}\right) \boldsymbol{x\ volume}\ (\boldsymbol{cm^3})$$

$$\boldsymbol{m_{NC}} = \boldsymbol{1.282x10^{-17}g}$$

(2)

$$\boldsymbol{N_{NC}} = \frac{\boldsymbol{m_{inorganic}}}{\boldsymbol{m_{NC}}}$$

$$\boldsymbol{N_{NC}} = \boldsymbol{1.95x10^{14}\ NCs}$$

(3)

$$\boldsymbol{mol\ of\ NC} = \frac{\boldsymbol{N_{NC}}}{\boldsymbol{N_A}}$$

$$\boldsymbol{mol\ of\ NCs\ (A) = 3.23\ x\ 10^{-10}\ mols}$$

(4)

$$\boldsymbol{mol}\,\% = \frac{\boldsymbol{A}}{\boldsymbol{A + mol\ reagent}}$$

$$\boldsymbol{mol}\,\% \approx \mathbf{0.001}\%$$

For the analysis of the material photoactivity was calculated the TON and TOF following the equation (5) and (6):

(5)

$$\boldsymbol{TON} = \frac{\boldsymbol{mol\ products}}{\boldsymbol{mol\ of\ NCs}}$$

$$\boldsymbol{TON} = \frac{3.4x10^{-5}}{3.23x10^{-10}} = \mathbf{105263}$$

(6)

$$\boldsymbol{TOF} = \frac{\boldsymbol{TON}}{\boldsymbol{time\ (h)}}$$

$$\boldsymbol{TON\ (h^{-1})} = \frac{105263}{48} = \mathbf{2192}$$

Modified Scherrer equation

The modified Scherrer equation method[4] has been used to get accurate NCs size, using the XRD pattern. The purpose of the modified Scherrer equation is to minimize the error of this equation, when the diameter of the NCs is measured at different XRD angles, using a least-squares technique. For that, it's plotted $\mathbf{ln}(\boldsymbol{\beta})$ (y - axis) against $\mathbf{ln}\left(\frac{\mathbf{1}}{\boldsymbol{cos\theta}}\right)$ (x - axis) obtaining an intercept of a least-squares line regression, which is referred to the diameter of the NCs.

For that, Scherrer equation (7) is modified to (8)

$$\beta = \frac{K\lambda}{Dcos\theta} \quad \textbf{(7)}$$

$$\ln(\beta) = \ln\left(\frac{1}{cos\theta}\right) + \ln\left(\frac{K\lambda}{D}\right) \quad \textbf{(8)}$$

**D**	grain size (nm)
**K**	0.9 (Scherrer constant)
**λ**	0.15406 nm (wavelength of the X-ray sources)
**β**	FWHM (in radians)
**θ**	peak position (in radians)

Then, the modified Scherrer equation could be plotted as a straight line (y=mx+c), and a least-square line regression is performed to calculate the grain size from the y-intercept "$\boldsymbol{c} = \ln\left(\frac{K\lambda}{D}\right)$" value.

$y = \ln(\beta)$ **(y - axis)**

$m = 1$ **(slope of line)**

$x = \ln\left(\frac{1}{cos\theta}\right)$ **(x - axis)**

$c = \ln\left(\frac{K\lambda}{D}\right)$ **(y intercept)**

- Calculation process:

a) Peak positions and FWHM of the XRD signals used for the calculations.

S. No	Peak Position	FWHM	ln(1/cosθ)	ln(β)
**1**	31.99579	0.45288	0.039498057	-4.840355054
**2**	39.45234	0.43117	0.060475876	-4.8894798
**3**	45.87559	0.46445	0.082372856	-4.815128334

b) Plott "$\ln\left(\frac{1}{cos\theta}\right)$ "against "$\ln(\beta)$" to obtain the intercept.

c) Calculation of the NCs size from the y-intercept:

$$\mathbf{-4.88533 = \ln\left(\frac{K\lambda}{D}\right)}$$

$$\boldsymbol{D = \frac{0.9 * 0.15406}{0.0075566} = 18.3\ nm}$$

Control experiments of the photocatalytic reduction of benzyl bromides

All the reactions have been performed in the same conditions reported for the photocatalytic reactions but in the presence of the scavengers. Regarding the control experiments, 0.34 mmol of TEMPO and $AgNO_3$ were added as scavengers, whereas the last reactions were performed in a normal air atmosphere. To avoid the presence of radicals produced after the toluene oxidation, all the reactions experiments were carried out in hexane and using Cl-benzyl bromide as the substrate to directly monitor the formation of the C-C coupling products.

Calculation of the photocatalytic activity in the recycling experiments

Recycling experiments were performed by triplicate. The NC dispersions were centrifuged after each photocatalytic cycle and the precipitate was reused in the next cycle. In this process, some amount of the photocatalyst was lost due to its adsorption to the glass reactor and/or during the precipitation step. To exclude the influence of the photocatalyst mass, the photocatalytic activity was calculated as follows: the product yield was divided by the photocatalyst mass for every single reaction and cycle, and then normalized by the maximum product yield (100%) and the initial amount of photocatalyst (10.5 mg in these reactions), using equation 9. As has been depicted in Figure S27a, the photocatalytic activity of the NCs remained very similar after the 3 cycles with a standard deviation between the different cycles of 5 %.

$$Photocatalytic\ activity = \frac{\left(\frac{Product\ yield\ (\%)}{Photocatalyst\ (mg)}\right) in\ each\ cycle}{\left(\frac{Max.\ Product\ yield\ (\%)}{Initial\ photocatalyst\ (mg)}\right)} \qquad \textbf{(9)}$$

Transmission Electron Microscopy (TEM) Analysis.

The samples were prepared by dropping dilute NC solutions onto carbon-coated 200 mesh copper grids. Low-resolution TEM analyses were performed on a HITACHI HT7800 microscope with a filament of LaB6 operated at 100 keV.

High resolution transmission Electron Microscopy (HRTEM) Analysis.

High-resolution TEM (HRTEM) images were taken on a Field Emission Gun (FEG) TECNAI G^2 F20 microscope operated at 200 kV. The samples were prepared by drop-casting few drops of a toluene NCs dispersion onto a carbon film supported on a copper grid, which was subsequently dried under vacuum before the examination.

Scanning Electron Microscopy and Energy-dispersive spectroscopy (SEM-EDS).

SEM-EDS analysis was performed on a HRSEM JEOL JSM-7500LA microscope with a cold field-emission gun (FEG), operating at 15 kV acceleration voltage. Energy-dispersive spectroscopy (EDS, Oxford instrument, X-Max, 80 mm2) was used to evaluate the elemental ratios. All experiments were done at an 8 mm working distance, 15 kV acceleration voltage, and 15 sweep count for each sample. SEM images were taken using a Hitachi S-4800, operating at 20 kV acceleration voltage.

Electrochemical properties of $Cs_3Sb_2Br_9$

Redox properties characterization was performed on an Autolab potentiostat (Autolab 128N potentiostat/galvanostat) using a three-electrode system. Cyclic voltammetry (CV) experiments were carried out in 0.1 M tetrabutylammonium tetrafluorophosphate ($TBAPF_6$) solution in dried acetonitrile (ACN) and a mixture of ACN:toluene (1:4 v/v) for the $Cs_3Sb_2Br_9$ NCs, respectively, by using a Pt working electrode, a Pt wire auxiliary electrode and a Ag/AgCl reference electrode separated from the tested solution by means of a Luggin capillary. The measurements were performed at room temperature (298 ± 1 K) partially deaerating the electrolyte solution by bubbling N_2 for 2 min. Experiments were performed under air conditions, with scan rates varying from 5 to 100 mV/s. To evaluate the electrochemical bandgaps, potentials were referred to the Fc/Fc^+ couple using 0.2 mM solutions of ferrocene in 0.1 M $TBAPF_6$ in their respective solvents.

For the calculation of the perovskite energy levels, the irreversible peak cathodic potential of $Cs_3Sb_2Br_9$ was considered since it was highly pronounced (-1.50 V vs. Ag/AgCl reference electrode, where there is a higher density of states). With the half-wave oxidation potential of the external standard Fc/Fc^+ (0.52 V vs. Ag/AgCl reference electrode) one can easily estimate the conduction band energy level of the $Cs_3Sb_2Br_9$ material as follows:

$$E_{CB} = -\left[V_{red} - V_{1/2}(Fc^+/Fc) + 4.8\right] \text{ eV} \qquad \textbf{(10)} \qquad \rightarrow \qquad \mathbf{E_{CB} = -2.78\ eV}$$

Since the oxidation peak potential of the $Cs_3Sb_2Br_9$ material was not as well resolved, the help of the conduction band energy and the optical bandgap (2.58 eV, calculated with Tauc plot equation; figure S.4 a) allowed us to roughly estimate the valence band energy level as follows:

$$E_{VB} = E_{CB} - \Delta E^{opt} \qquad \textbf{(11)} \qquad \rightarrow \qquad \mathbf{E_{VB} = -5.36\ eV}$$

Note that if the oxidation potential of the $Cs_3Sb_2Br_9$ material is taken as 1 V (second irreversible peak at positive CV values), the electrochemical gap (2.5 eV) coincides approximately with the value of the optical gap (2.58 eV). This suggests that this is an acceptable approach for estimating energy levels. This methodology is widely used in literature to determine the band gap of semiconductors.[5]

2. Supporting tables

Table S1: Energy Dispersive X-Ray Analysis (EDAX) obtained from the average of two samples.

ANALYSIS	ELEMENT	ATOMIC %	MOLAR RATIO (X/Cs)
1	Cs	19.91	2.5
	Sb	16.12	2
	Br	63.85	8

Table S2: Screening of different conditions for optimization.

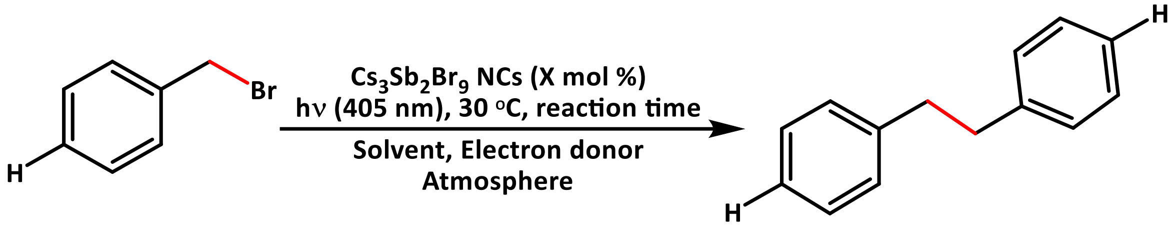

Entry	Variance	Conversion (%)	1a (%)
**Electron donor**			
**1**	Diisopropylethylamine (DIPEA)	0	0
**2**	Methanol (MeOH)	21	11
**Amount of electron donor - MeOH (eq.=equivalents[a])**			
**3**	89 mM (5.23 eq.)	18	10
**4**	340 mM (20 eq.)	99	32
**Amount of substrate**			
**5**	17 mM	99	32
**6**	65 mM	75	13
**Reaction time**			
**7**	8 h	45	16
**8**	24 h	67	15
**9**	48 h	80	19
**Gas atmosphere**			
**10**	Oxygen	Traces	2
**11**	Argon	88	72
**12**	Nitrogen (glovebox)	100	82
**Amount of photocatalyst**			
**13**	0.001 mol %	93	87
**14**	0.003 mol %	100	91

Reaction conditions: benzyl bromide (X mM), $Cs_3Sb_2Br_9$ NCs (X mol %), electron donor (X mM) in toluene (2 mL), X h irradiation at = 405 and 30 °C under different atmospheres. Note: All the yields are determined by GC-MS using biphenyl (75 mM) as internal standard. [a] Equivalents are calculated compared to the amount of substrate. Average from triplicate measurements

Table S3. Retention time (RT) of the different analytes studied

RT (min)	Compound
**Common analytes observed**	
5.438	*p*-Xylene – impurity of the toluene used for injecting samples in the GC
15-264-15.270	Biphenyl – internal standard
18.634-18.640	Dioctylether - residues of the NCs synthesis
20.038-20.044	Octadecene – residues of the NCs synthesis
**Substrate: Benzyl bromide**	
17.114	Bibenzyl
**Substrate: *p-tert*-Butylbenzyl bromide**	
10.593	4-tert-Butyltoluene
23.738	4,4'-Di*tert*-butylbibenzyl
20.749	4-*tert*-butylbibenzyl
17.107	Bibenzyl
**Substrate: *p*-Methoxybenzyl bromide**	
9.112	4-Methoxyltoluene
22.748	4,4'-Dimethoxybibenzyl
20.093	4-Methoxybibenzyl
13.200	4-methoxybenzyl methyl ether
17.107	Bibenzyl
**Substrate: *p*-Bromobenzyl bromide**	
9.604	4-Bromotoluene
23.912	4,4'-Dibromobibenzyl
20.646	4-Bromobibenzyl
27.101	Bibenzyl
**Substrate: *p*-Chlorobenzyl bromide**	
7.644	4-Chlorotoluene
21.868	4,4'-Dichlorobibenzyl
19.546	4-Chlorobibenzyl
17.101	Bibenzyl
**Substrate: *p*-Nitrobenzyl bromide**	
17.495	*p*-Nitrobenzyl bromide

Table S4: Analysis of the toluene oxidation in the reactions performed in Table 2.

Entry	R	1b (%)*
**1**	OCH_3	0.0084
**2**	^{t}Bu	0.0075
**3**	H	0.0063
**4**	Br	0.0094
**5**	Cl	0.0260

*Product yield calculated concerning the initial amount of toluene (18.8 mmol)

3. Supporting figures

3.1. Photocatalyst characterization

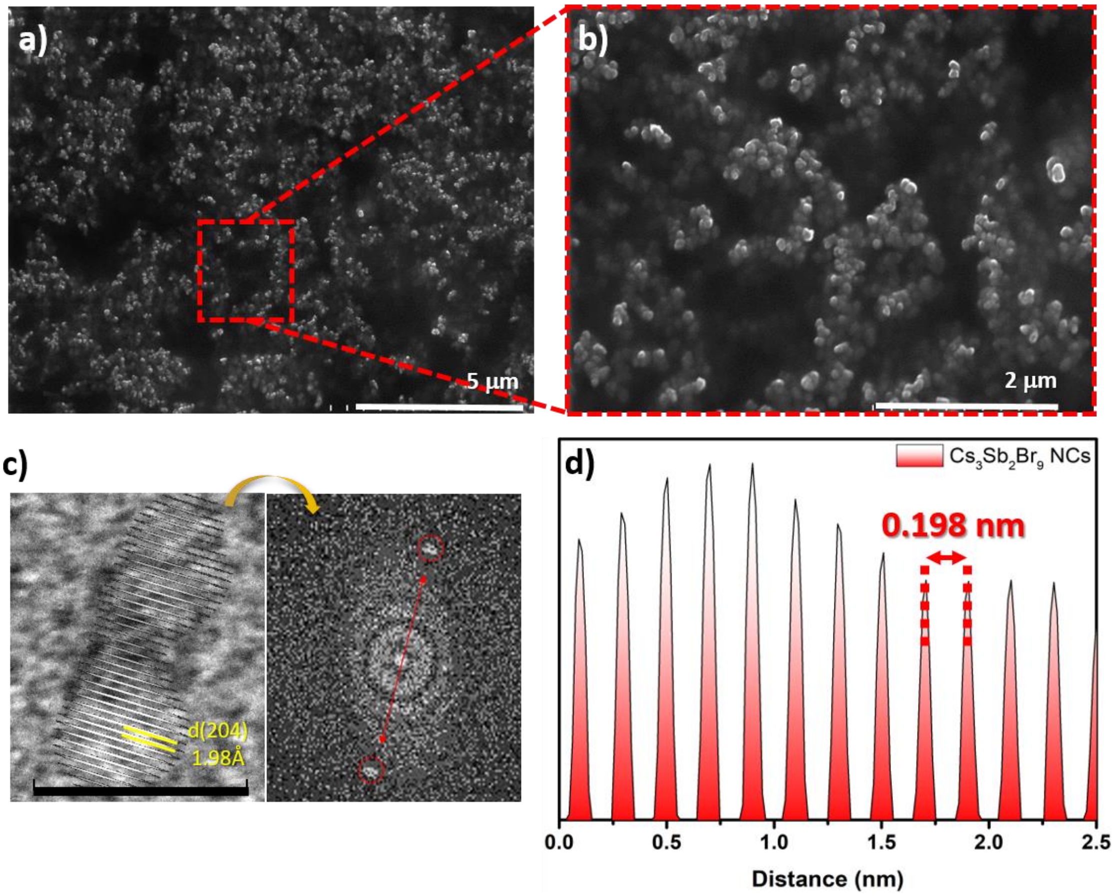

Figure S1. a-b) Representative SEM images of the spheric $Cs_3Sb_2Br_9$ NCs. c) HRTEM of one $Cs_3Sb_2Br_9$ NC overlapped with its inverted Fourier transformation (scale bar 10 nm) and d) intense height profile of the planes measured disclosing the presence of an interplanar distance of 0.198 Å which is ascribed to the (204) crystalline plane.

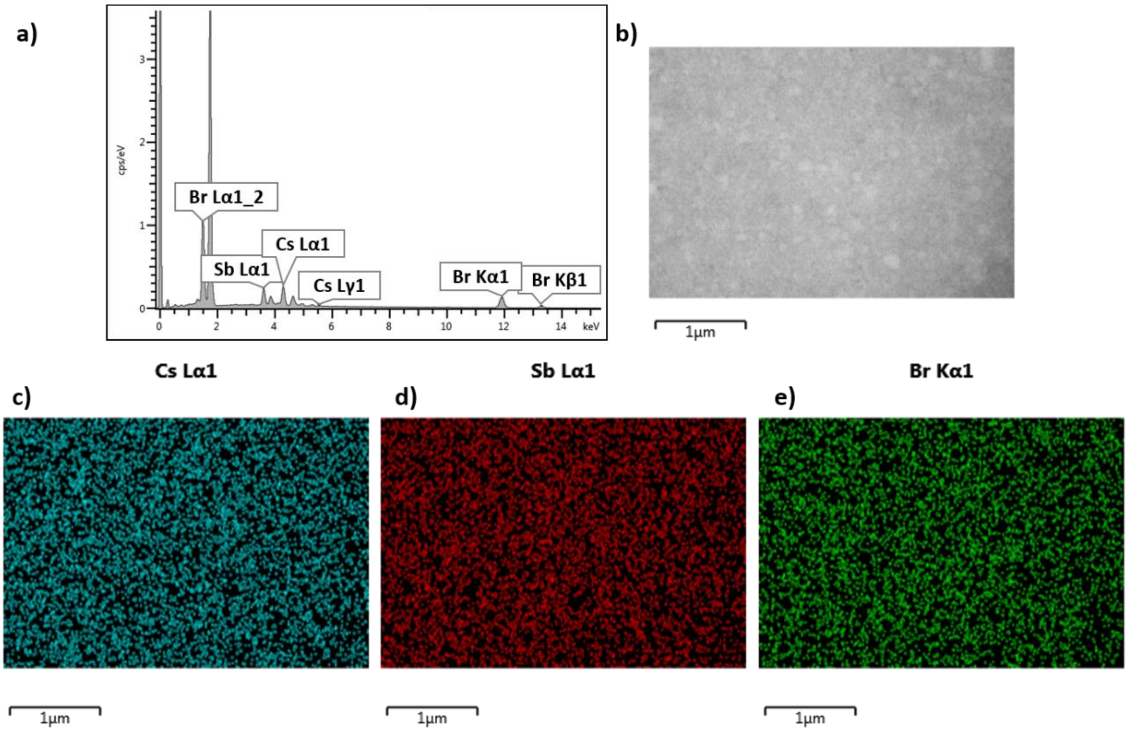

Figure S2. a) EDS spectrum collected for b) the SEM image of $Cs_3Sb_2Br_9$ NCs, and their corresponding mapping for c) Cs, d) Sb and e) Br elements.

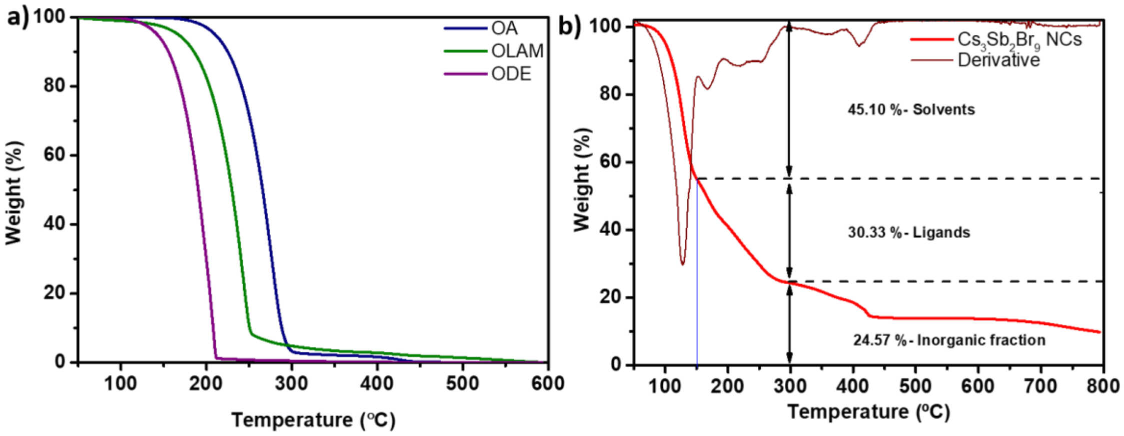

Figure S3. Thermogravimetric analysis of a) OA, OLAM, ODE and b) $Cs_3Sb_2Br_9$ NCs.

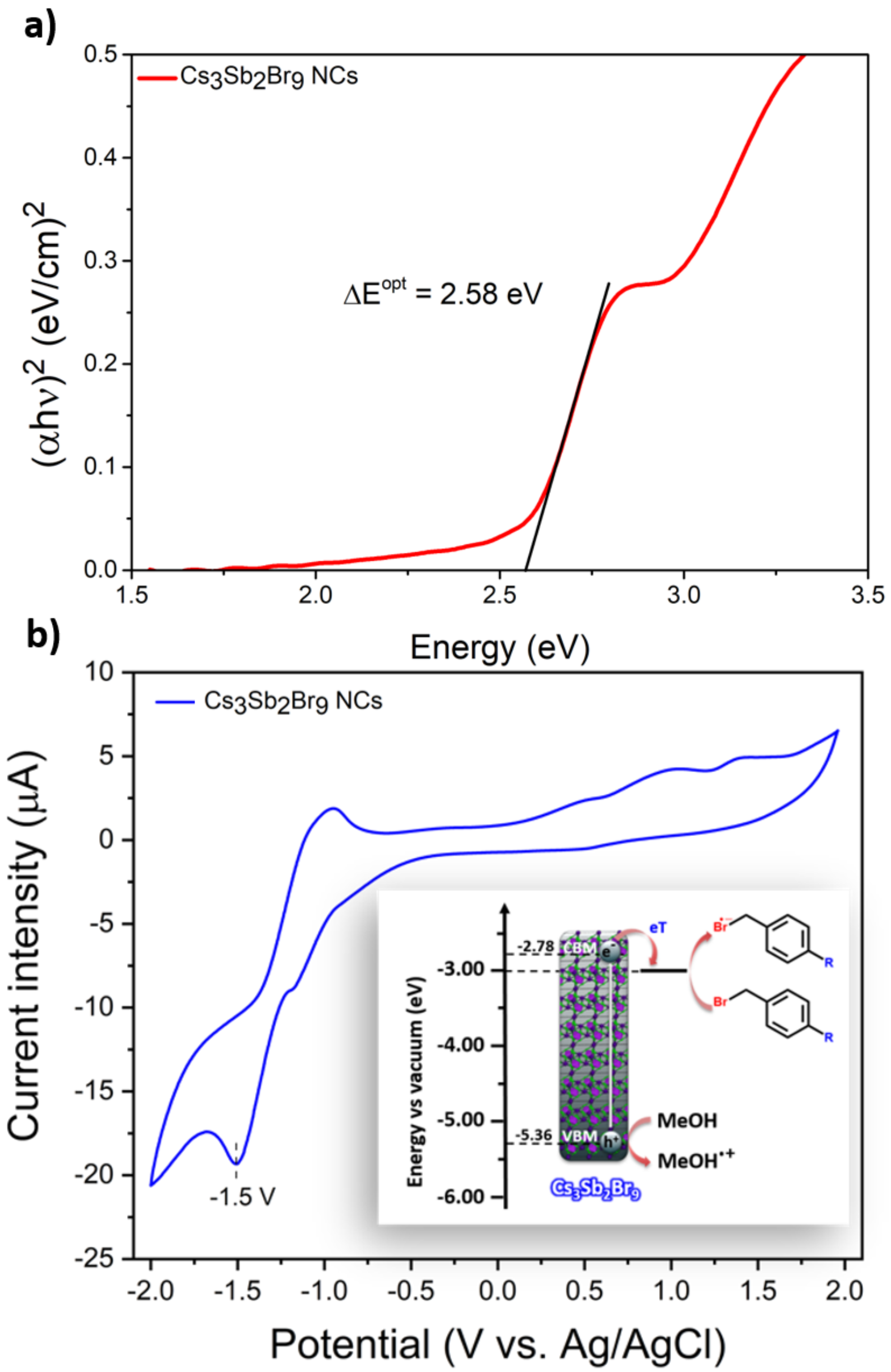

Figure S4. a) Absorption spectrum of $Cs_3Sb_2Br_9$ NCs. Inset: The straight line indicates the optical bandgap determined from Tauc plot. b) CV curve of the $Cs_3Sb_2Br_9$ NCs with an inset of the eT process from the NCs to the benzyl bromides.

3.2 Control experiments of the toluene oxidation

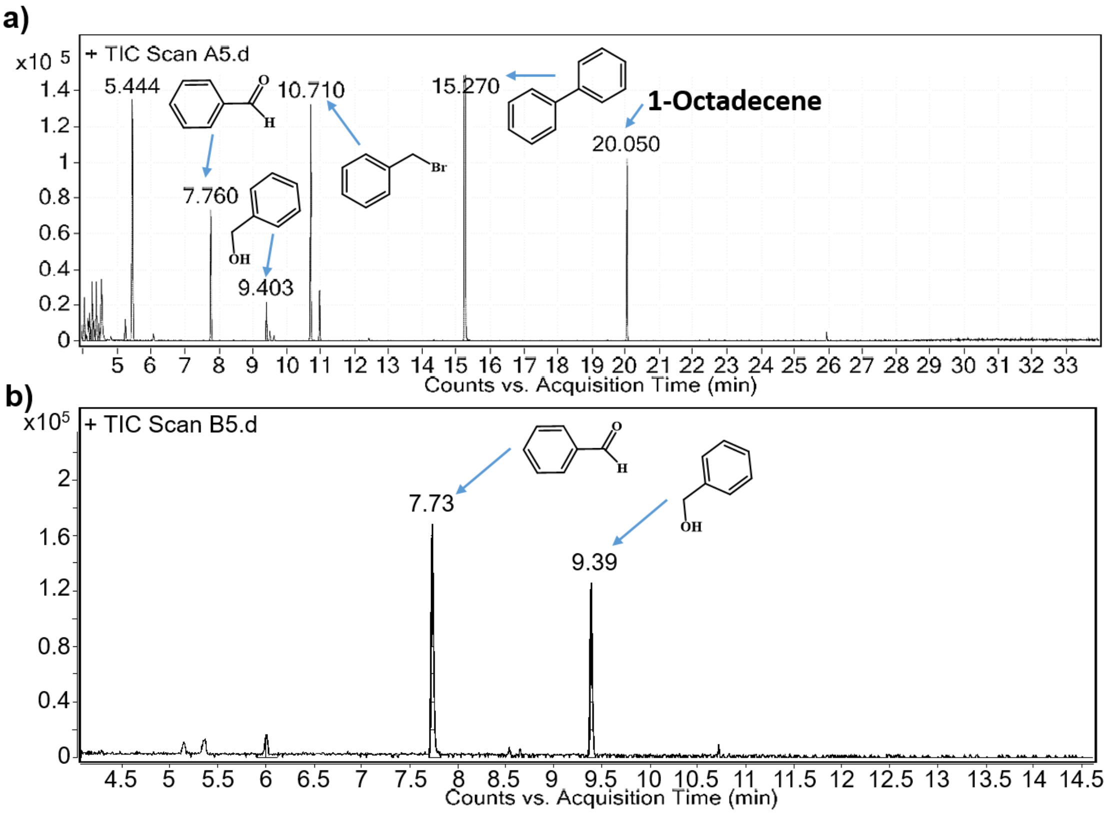

Figure S5. Gas chromatography of control reactions containing $Cs_3Sb_2Br_9$ NCs a) in the presence and b) absence of **1a** in toluene and purged with O_2. The signal at 5.44 and 15.25 min of retention time comes from the residual solvent (xylene) used for cleaning the GC-column after reactions and the biphenyl used as internal standard, respectively.

3.3 Products characterization

For product characterization, gas chromatography coupled with mass spectrometry was used. In these studies, biphenyl (75 mM) was employed as an internal standard. Table S3 summarizes the retention time of the different analytes observed.

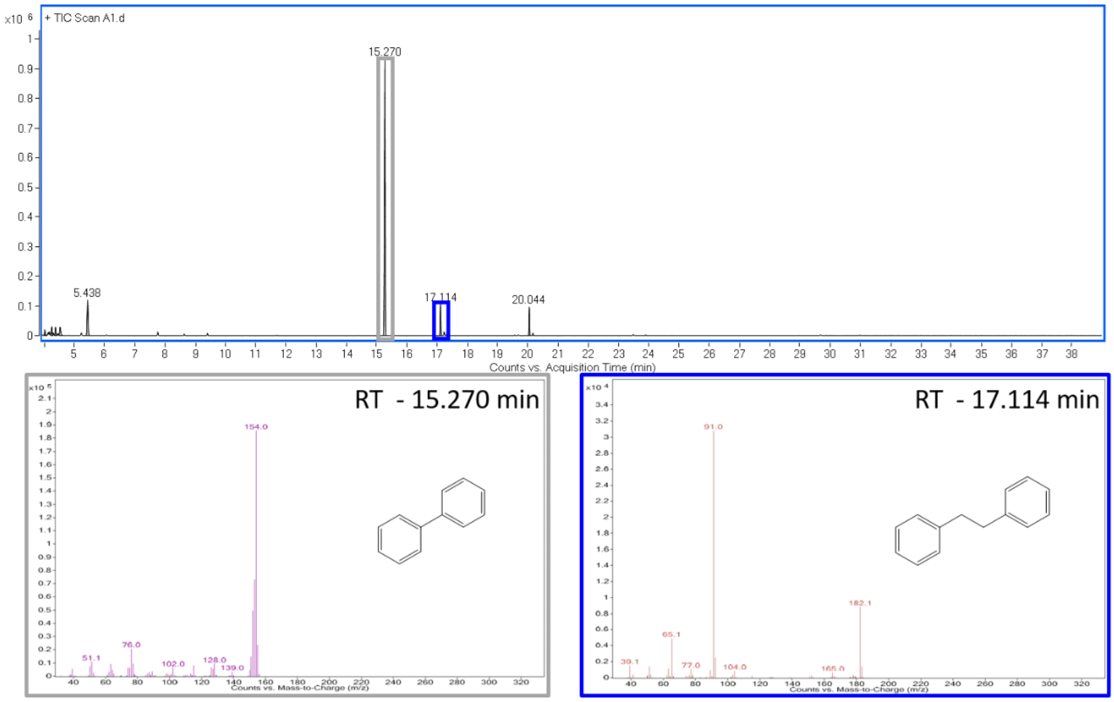

Figure S6. Gas chromatography of the benzyl bromide reaction in toluene and mass spectra of the biphenyl internal standard and bibenzyl product.

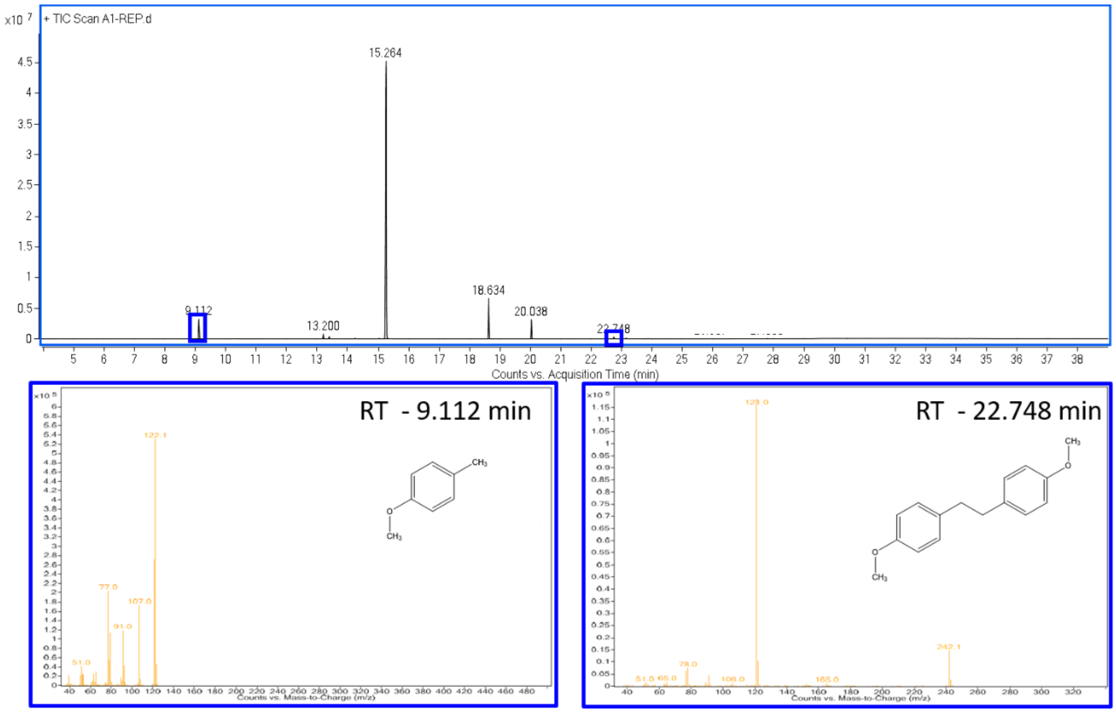

Figure S7. Gas chromatography of the *p*-OMe-benzyl bromide reaction in hexane and mass spectra of the products.

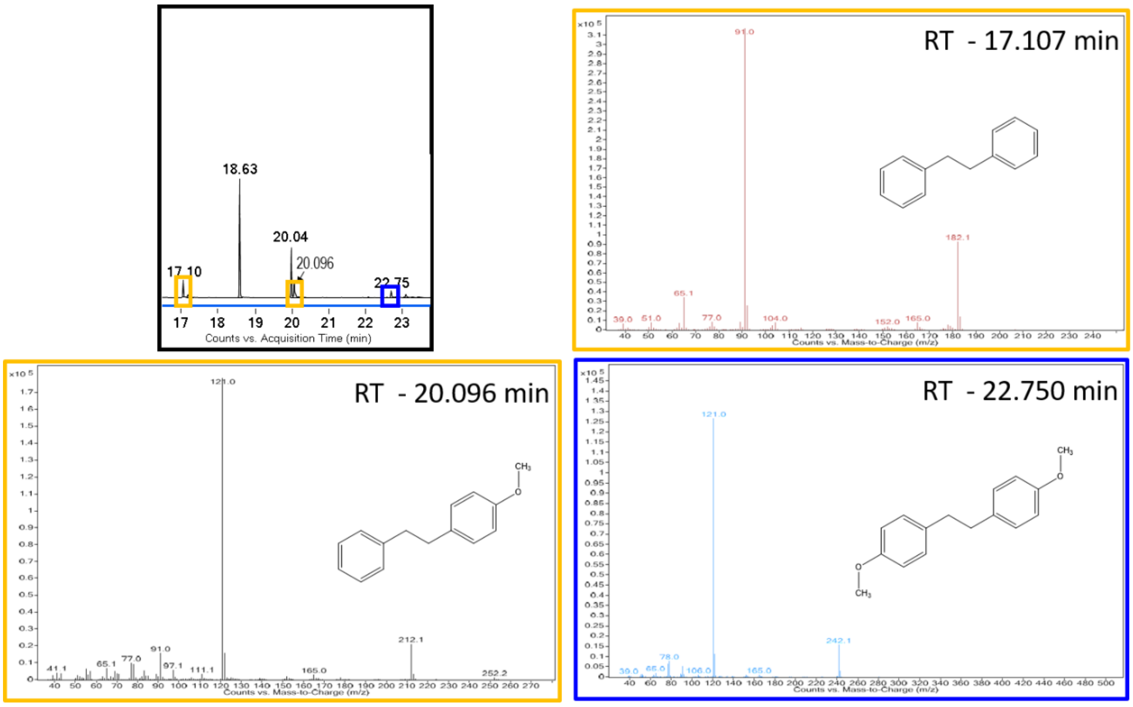

Figure S8. Gas chromatography of the *p*-OMe-benzyl bromide reaction in toluene and mass spectra of the products.

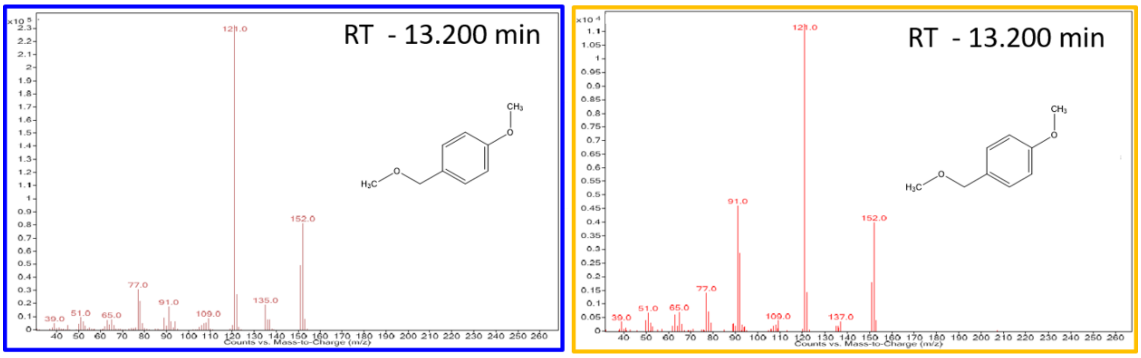

Figure S9. Mass spectra of the *p*-methoxybenzyl methyl ether in hexane (blue) and toluene (orange).

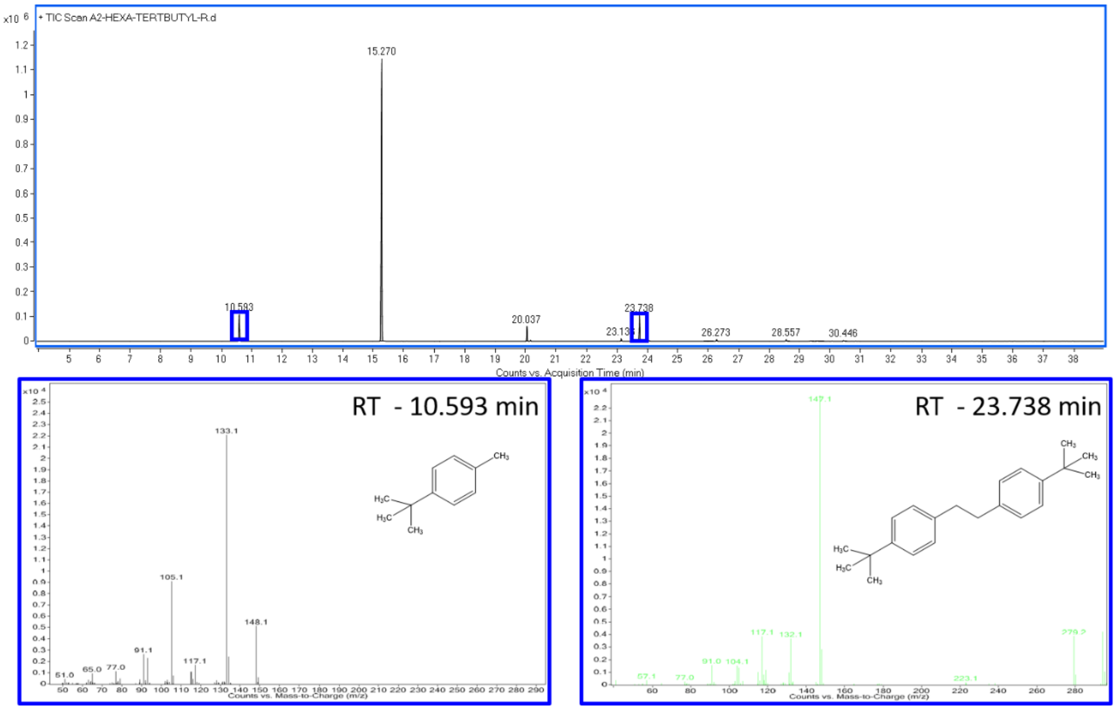

Figure S10. Gas chromatography of the *p*-^{t}Bu-benzyl bromide reaction in hexane and mass spectra of the products.

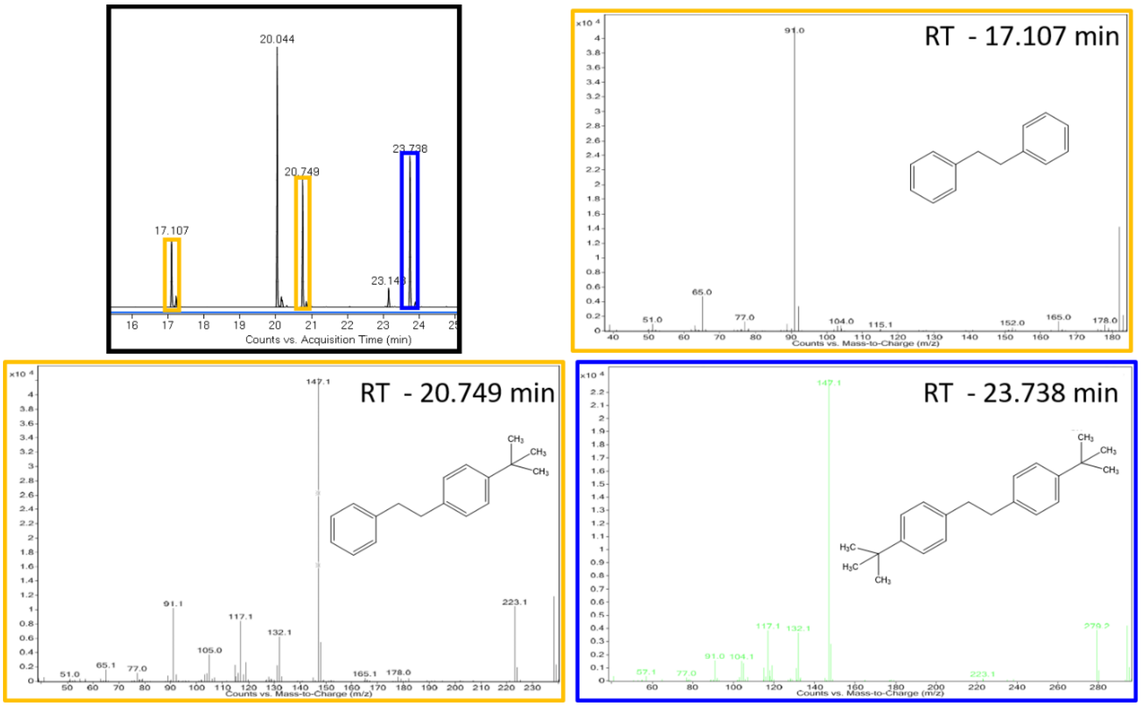

Figure S11. Gas chromatography of the *p*-^{t}Bu-benzyl bromide reaction in toluene and mass spectra of the products.

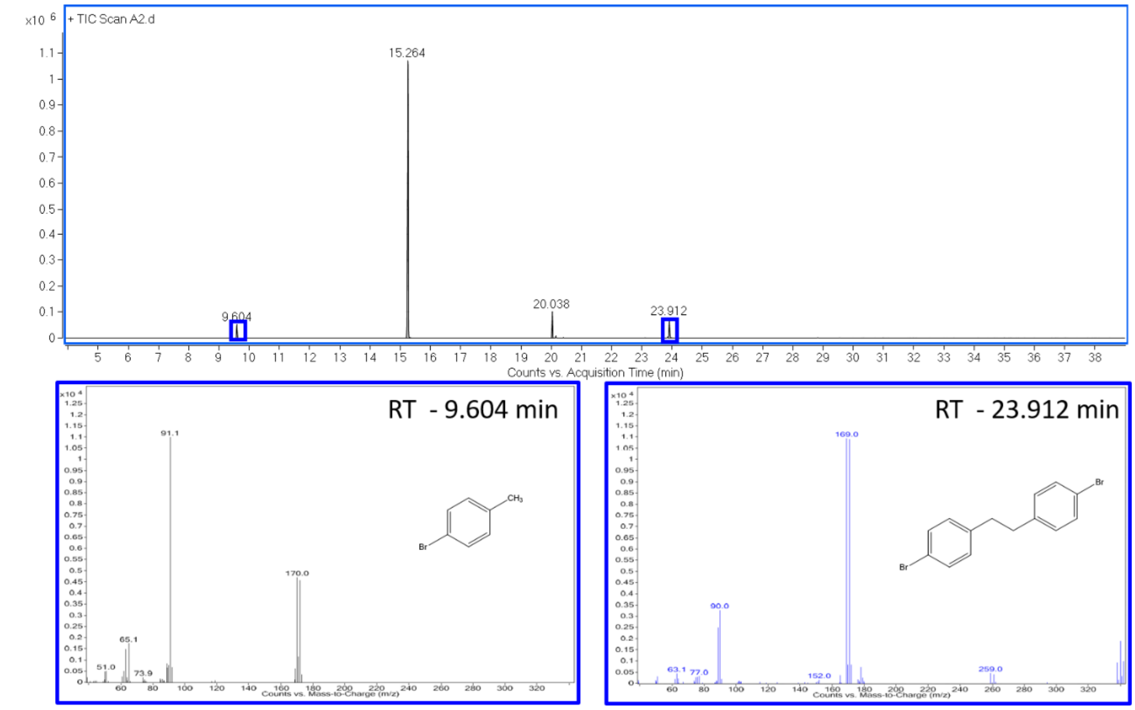

Figure S12. Gas chromatography of the *p*-Br-benzyl bromide reaction in hexane and mass spectra of the products.

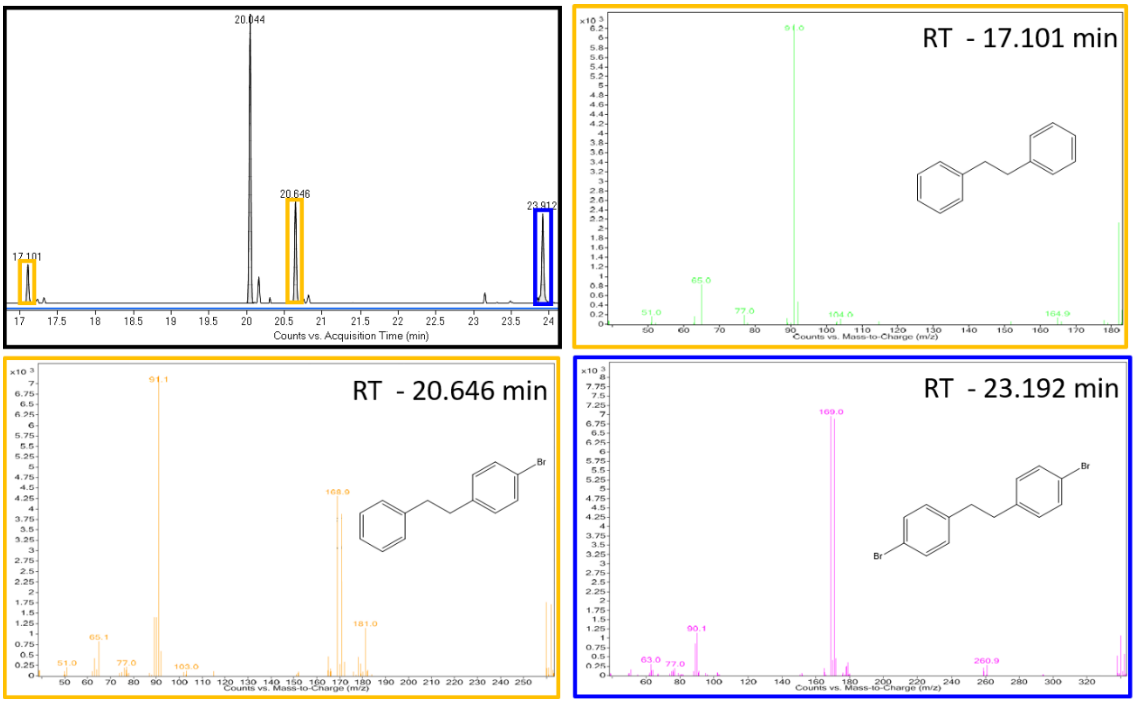

Figure S13. Gas chromatography of the *p*-Br-benzyl bromide reaction in toluene and mass spectra of the products.

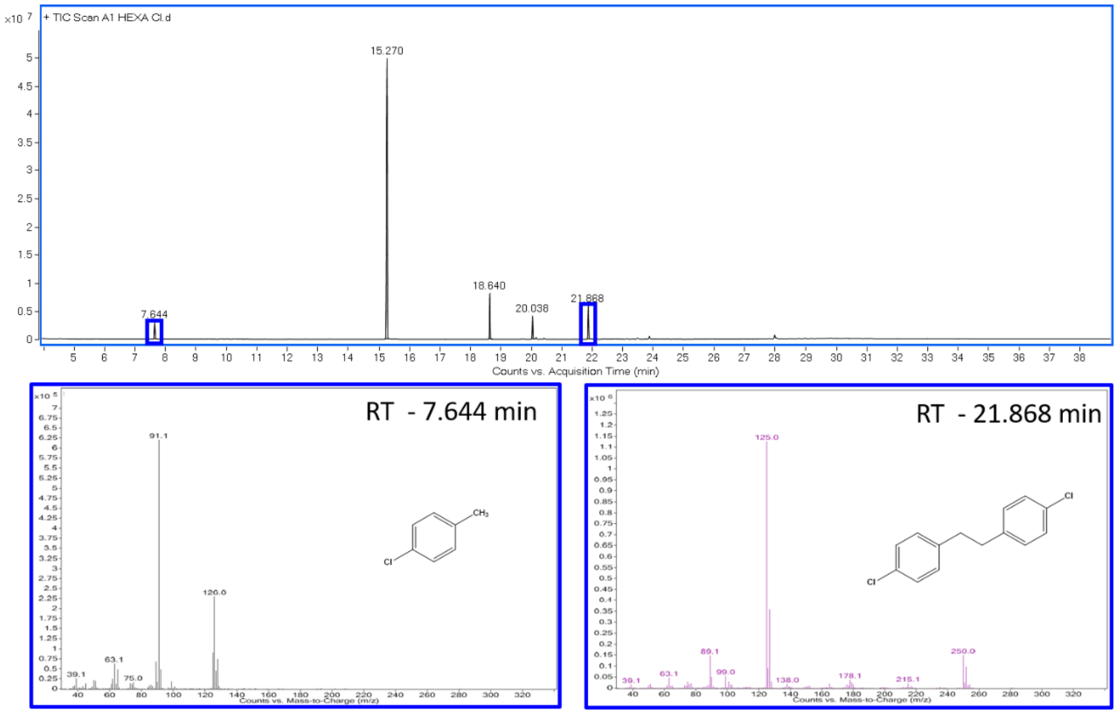

Figure S14. Gas chromatography of the *p*-Cl-benzyl bromide reaction in hexane and mass spectra of the products.

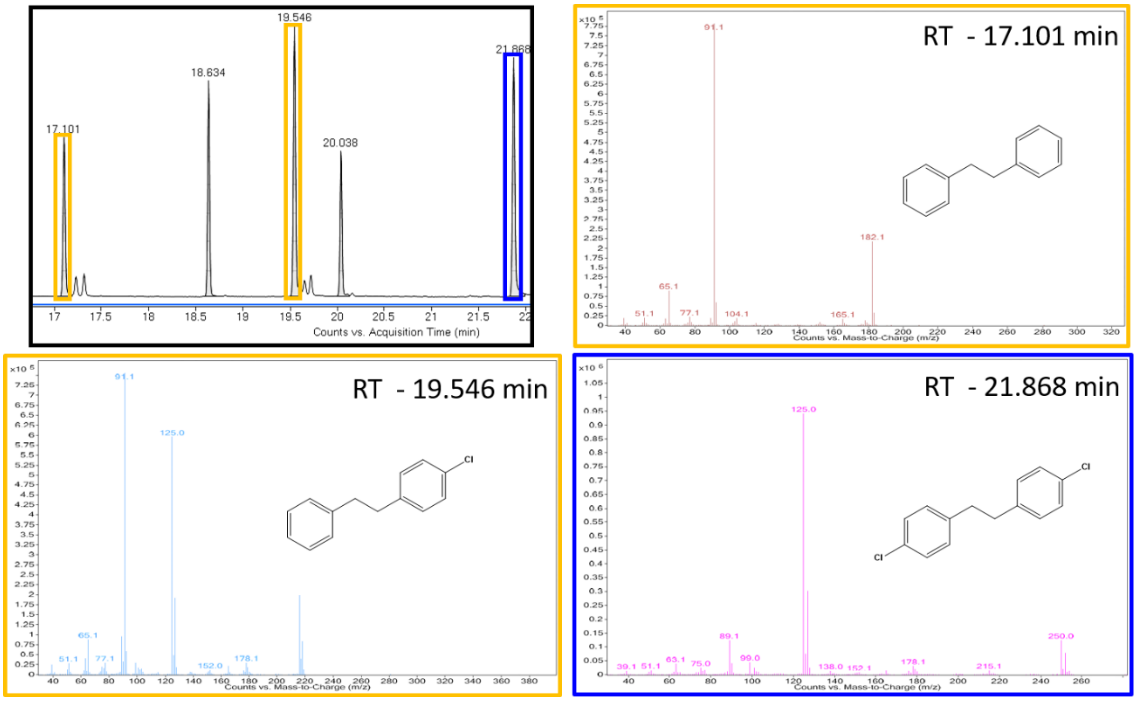

Figure S15. Gas chromatography of the *p*-Cl-benzyl bromide reaction in toluene and mass spectra of the products.

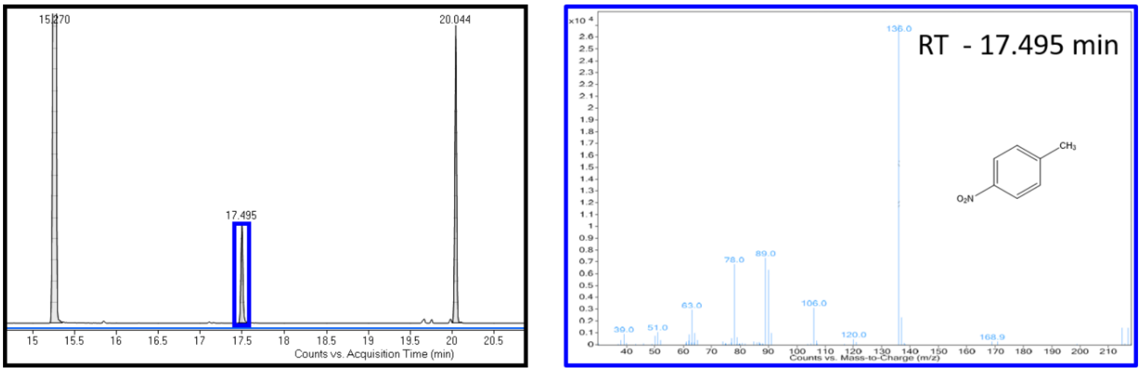

Figure S16. Gas chromatography of the *p*-NO_2-benzyl bromide reaction in toluene and mass spectra of the substrate.

3.5. Mechanistic studies of the photocatalytic process

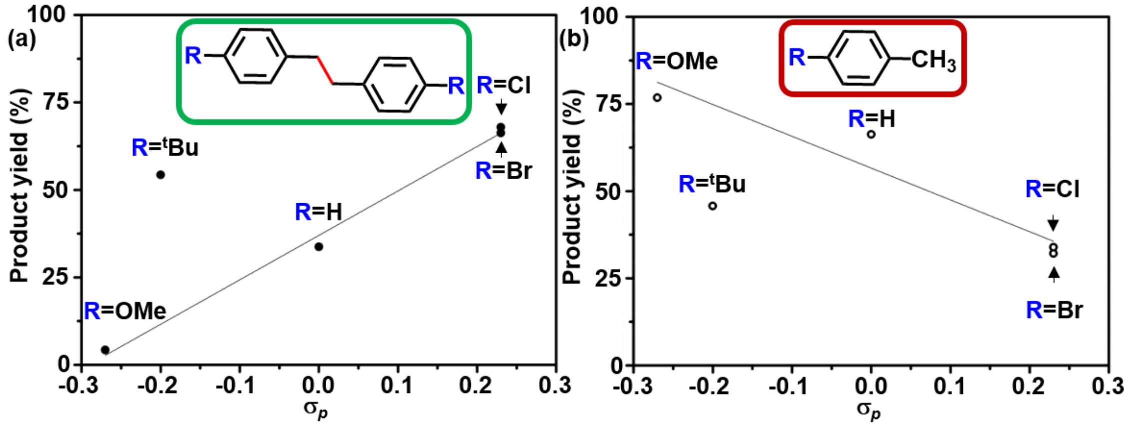

Figure S17. Correlation between the product yield of the C-C homocoupling product (a) and that of the dehalogenation product (b) with the sigma value (σ_p) for different *p*-substituted benzyl bromides.

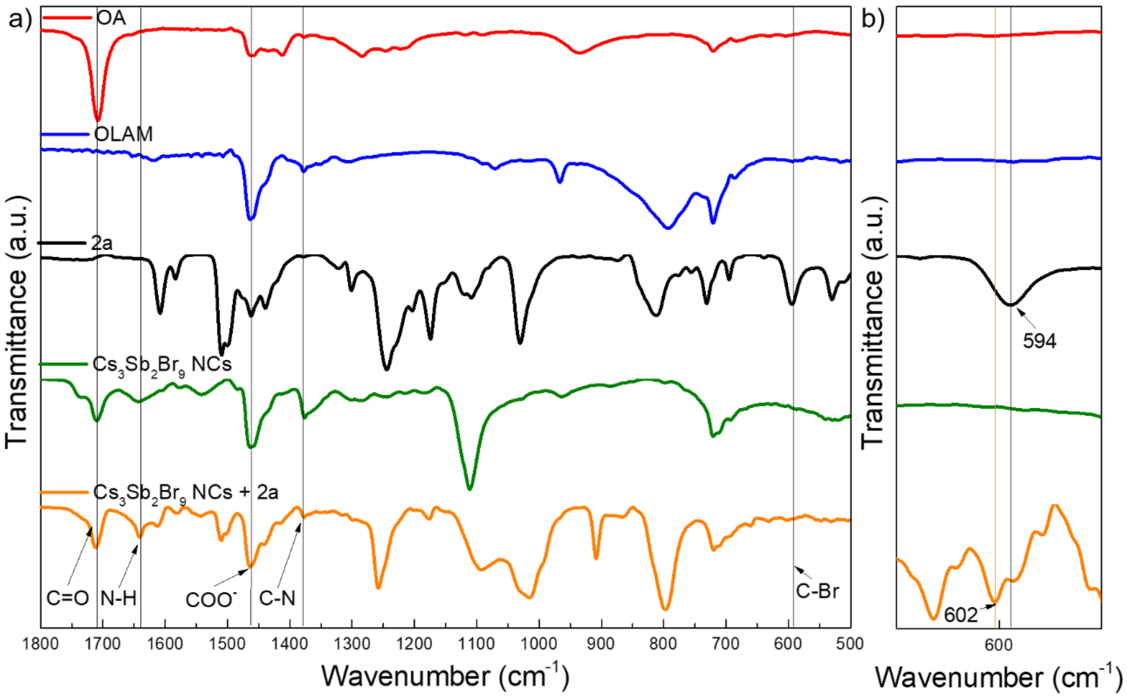

Figure S18. a) FTIR spectra of OA (red line), OLAM (blue line), 2a (black line), $Cs_3Sb_2Br_9$ NCs (green line) and $Cs_3Sb_2Br_9$ NCs with 2a (orange line). b) FTIR zoom of the C-Br stretch region.

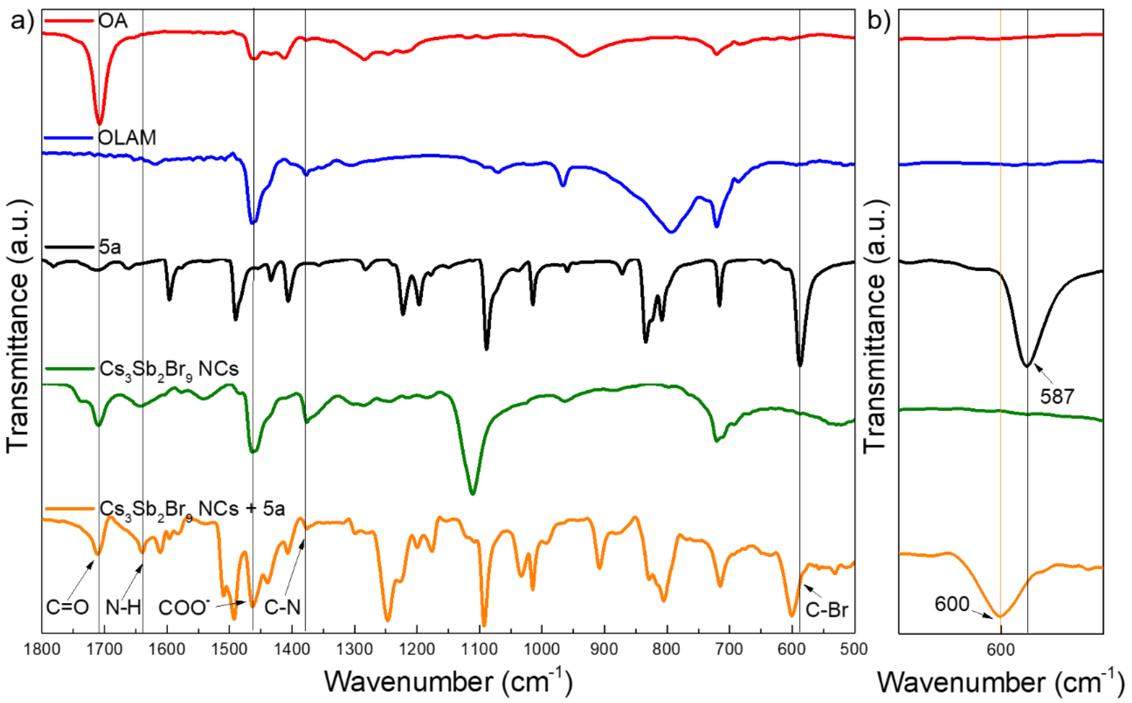

Figure S19. a) FTIR spectra of OA (red line), OLAM (blue line), **5a** (black line), $Cs_3Sb_2Br_9$ NCs (green line) and $Cs_3Sb_2Br_9$ NCs plus **5a** (orange line). b) FTIR zoom of the C-Br stretch region.

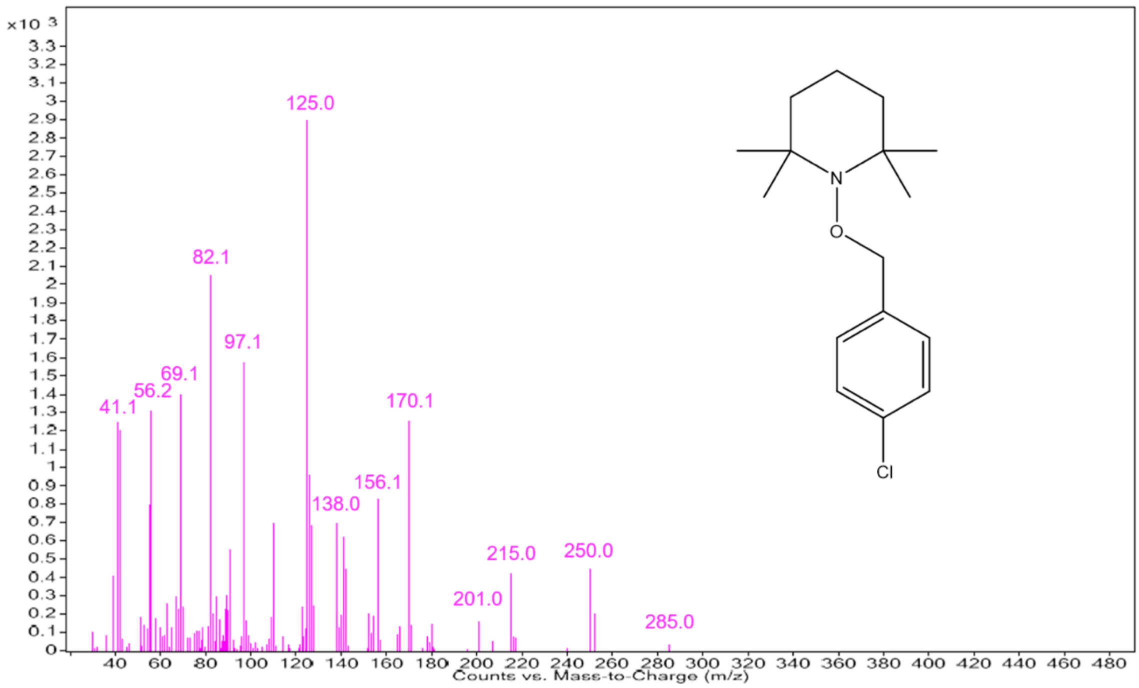

Figure S20. Mass spectrum of the radical-TEMPO adduct.

3.6. Br_2 detection experiments

With the aim of ascertaining if the Br_2 content could come from the leaching of the structural Br from $Cs_3Sb_2Br_9$ NCs under the photocatalytic reaction conditions, control experiments were carried out to study the Br_2 formation. The reaction was performed in the presence and the absence of benzyl bromide (**1a**), preserving the same photocatalytic conditions as those used in all the reactions performed before. As can be observed in Figure 5, the supernatant of the reaction in the presence of substrate presented a brownish color and the absorption spectra showed the typical band ($\lambda_{abs.\ max.}$=324 nm) ascribed to the charge-transfer complex between the Br_2 and aromatic compounds. However, when no substrates were added to the reaction, the supernatant was colorless, thus ruling out the possible loss of Br- from the NC surface.

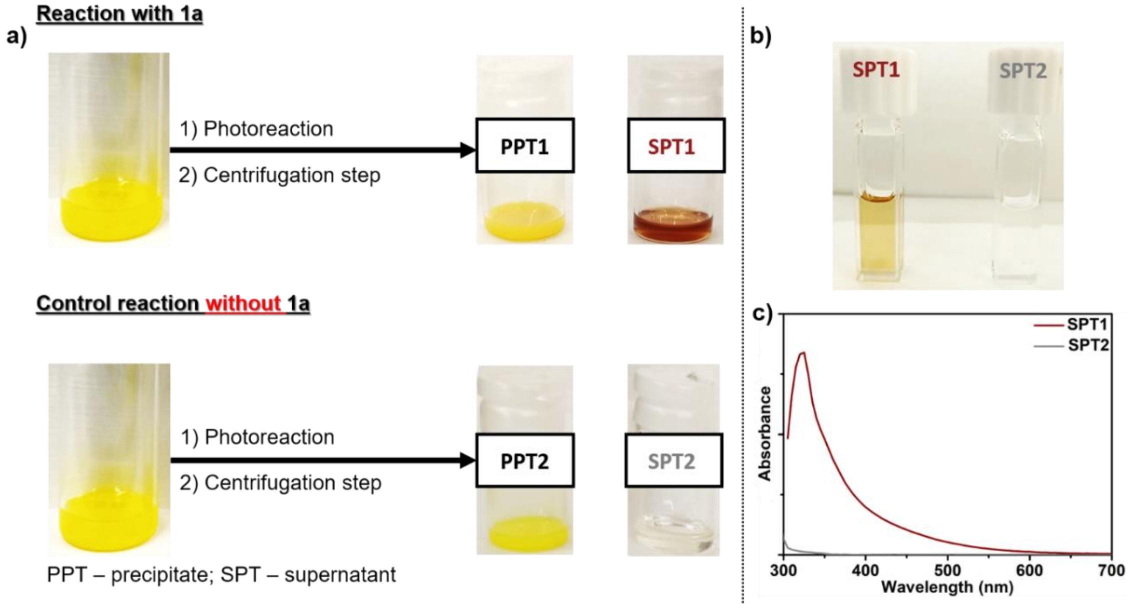

Figure S21. a) Schematic process of the reactions performed in the presence and absence of benzyl bromide (1a) together with the b) the image taken to the quartz cuvettes with the supernatants and the UV-VIS spectra of them.

The addition of cyclohexene in carbon tetrachloride (CCl_4) to the brown-yellowish supernatant of the *p*-OMe-benzyl bromide reaction did not produce any discoloration associated with the addition of the Br_2 to the double bound.[6-8] As, the direct measurement of molecular Br_2 was not possible because it formed a charge transfer complex with the aromatic compounds (substrate and products), as it has been previously reported for Br_2-benzene complexes (absorption band ca. 300 nm, Figure S16).[9-11] Two additional experiments were done to confirm the presence of molecular Br_2: i) the addition of KOH to break down the charge transfer complexes, followed by UV-light irradiation to deliver molecular Br_2 (Figure S17a, inset molecular Br_2 spectrum) and ii) irradiation of the charge transfer complex (λ_{ex}=365 nm during 90 minutes), thus eventually leading to the substitution of H by Br with the concomitant discoloration of the sample (Figure S17b). The photoaddition of Br_2 to aromatic compounds has been reported for bromobenzenes and *p*-substituted toluene derivatives.[12, 13]

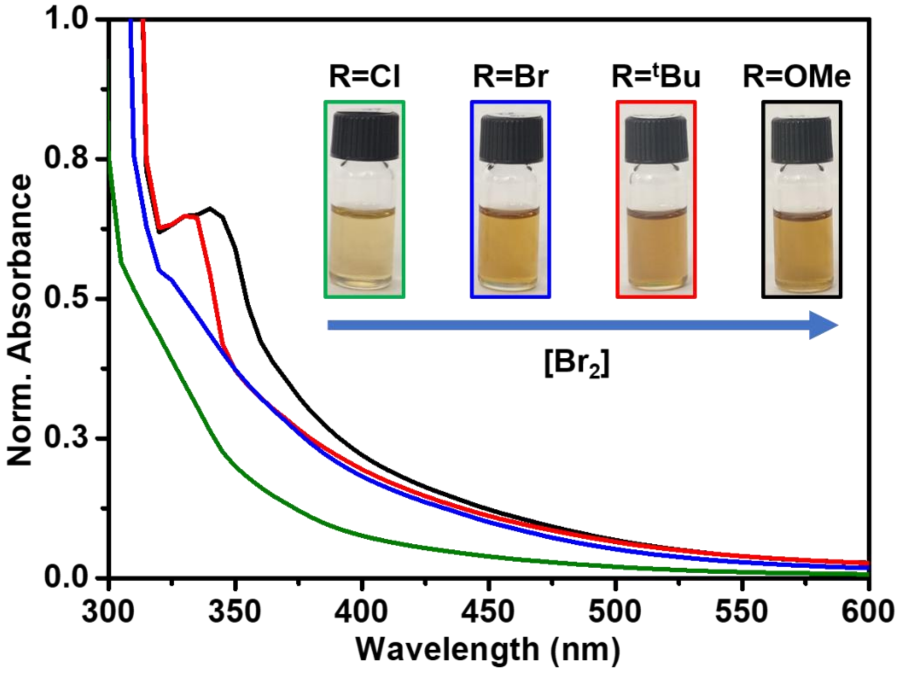

Figure S22. UV−vis absorption spectra of the supernatant after the photocatalytic photoreduction of *p*-substituted benzyl bromides **5a**, **4a**, **3a** and **2a** (R= Cl, Br, ^{t}Bu and OMe) in toluene.

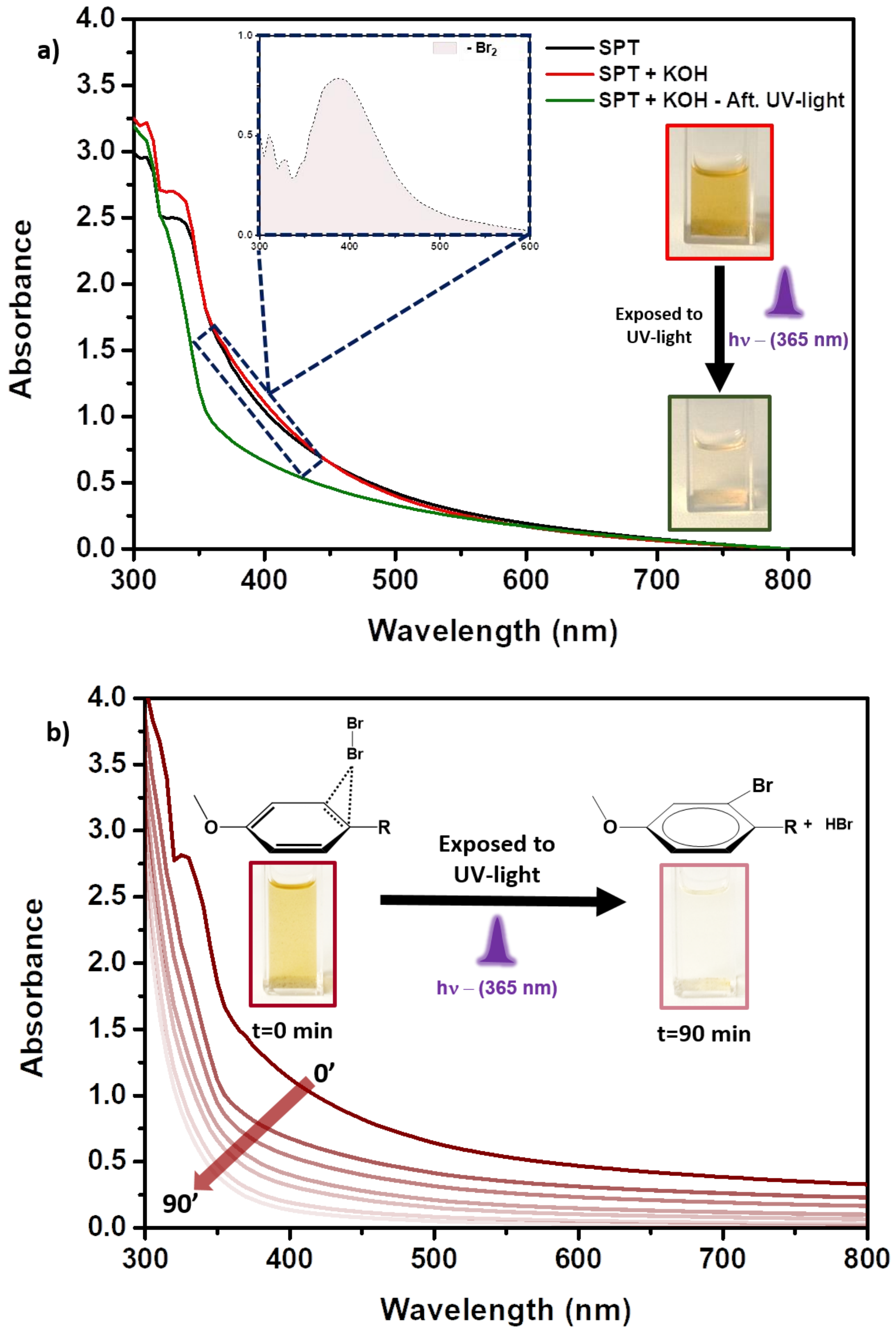

Figure S23. UV–vis absorption spectra of the supernatant (SPT) after one photocatalytic photoreduction of **2a** in toluene. Both experiments were performed in isopropyl alcohol by removing the toluene under vacuum followed by a) the addition of KOH or b) direct irradiation at 365 nm to study the disappearance of the Br_2 absorption band due to the photoaddition of Br to the aromatic ring.[12, 13]

3.7. Photocatalyst performance after photocatalytic cycles

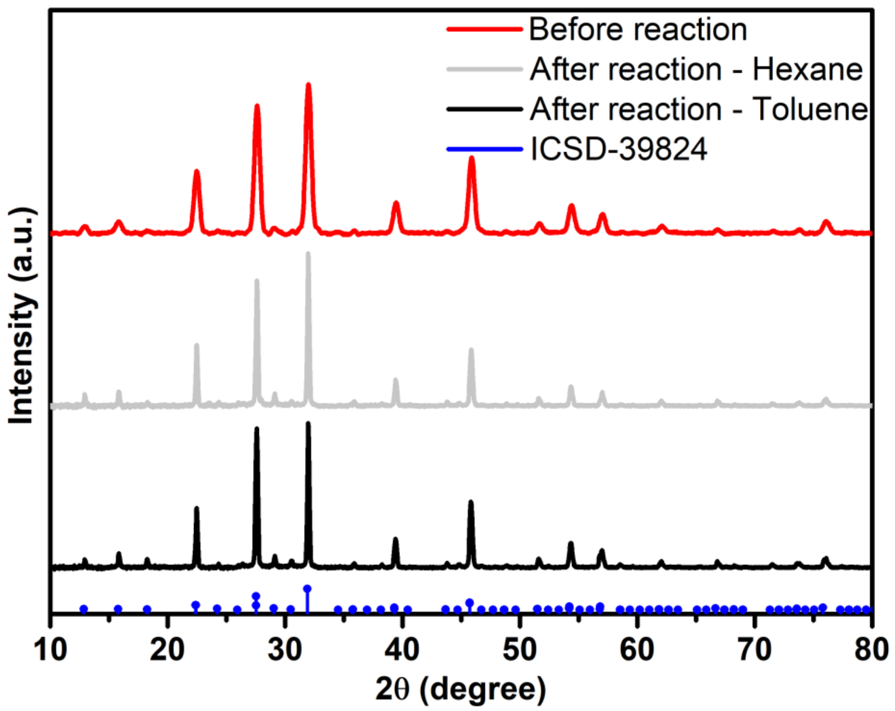

Figure S24. XRD patterns of $Cs_3Sb_2Br_9$ NCs sample before (red) and after one photocatalytic reaction with benzyl bromide **1a** in (grey) hexane and (black) toluene. In blue, hexagonal $Cs_3Sb_2Br_9$ crystal structure (ICSD number 39824).

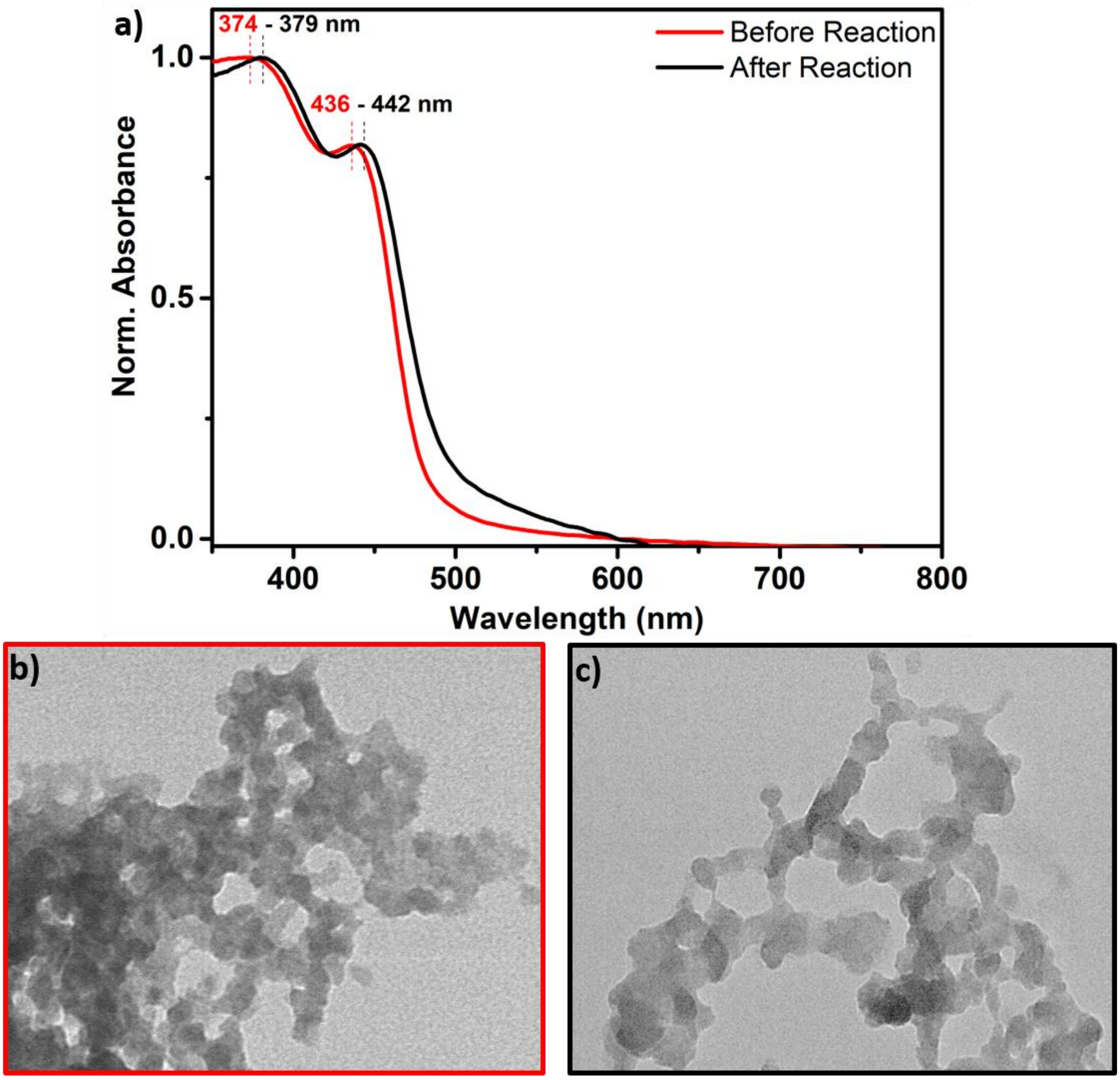

Figure S25. a) UV–vis absorptions spectra of $Cs_3Sb_2Br_9$ NCs films measured in an integration sphere (black line) before and (red line) after one photocatalytic cycle with benzyl bromide **1a.** TEM images b) before and c) after the photocatalytic cycle.

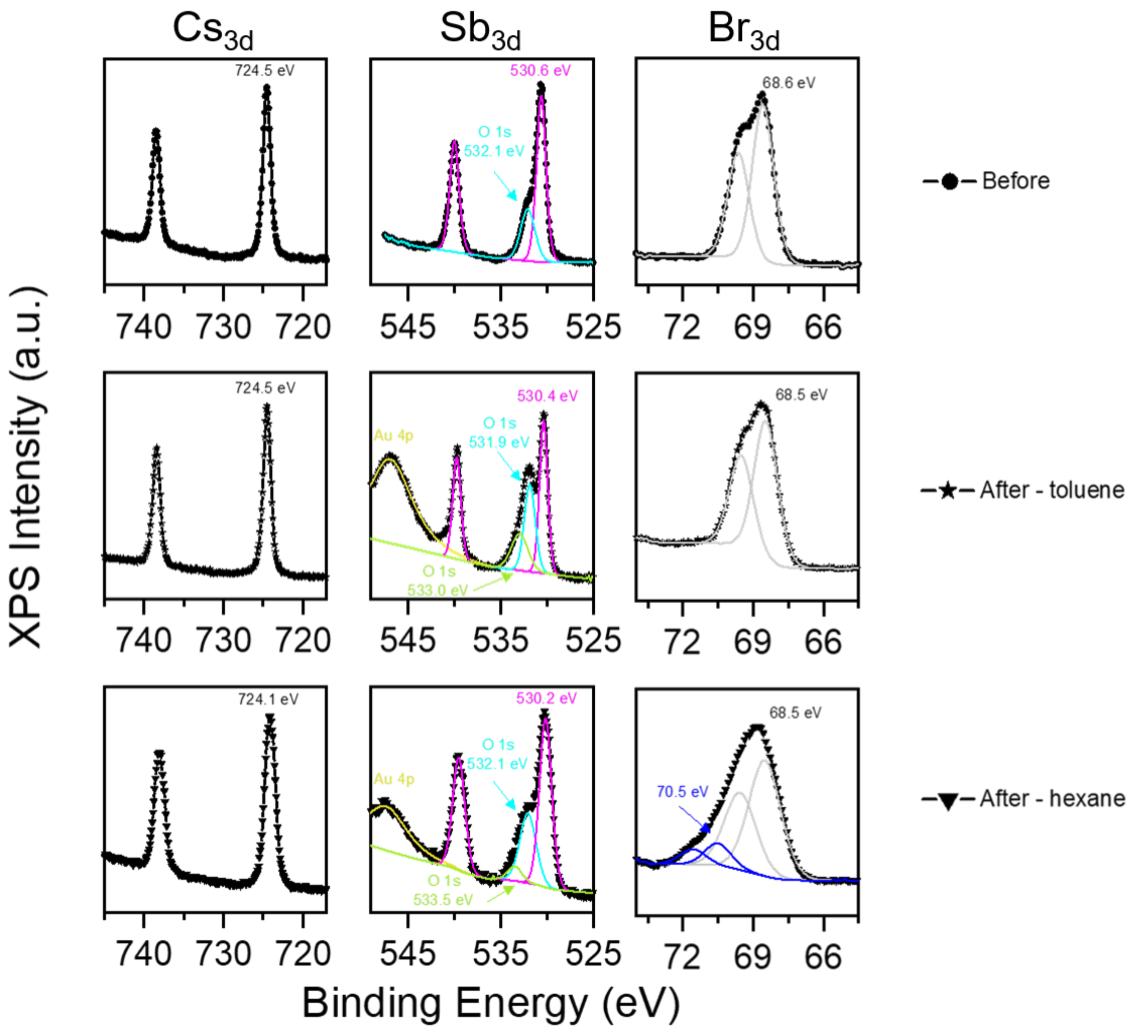

Figure S26. XPS spectra for $Cs_3Sb_2Br_9$ NCs (first row – circle dot) before and after the photoreduction of with *p*-Br-benzyl bromide **4a** performed (second row – star dot) in toluene or (third row - triangle dot) in hexane of Cs_{3d}, Sb_{3d}, and Br_{3d}. The Au 4p band ca. 542 ev observed after the photocatalytic cycle corresponds to the substrate used.

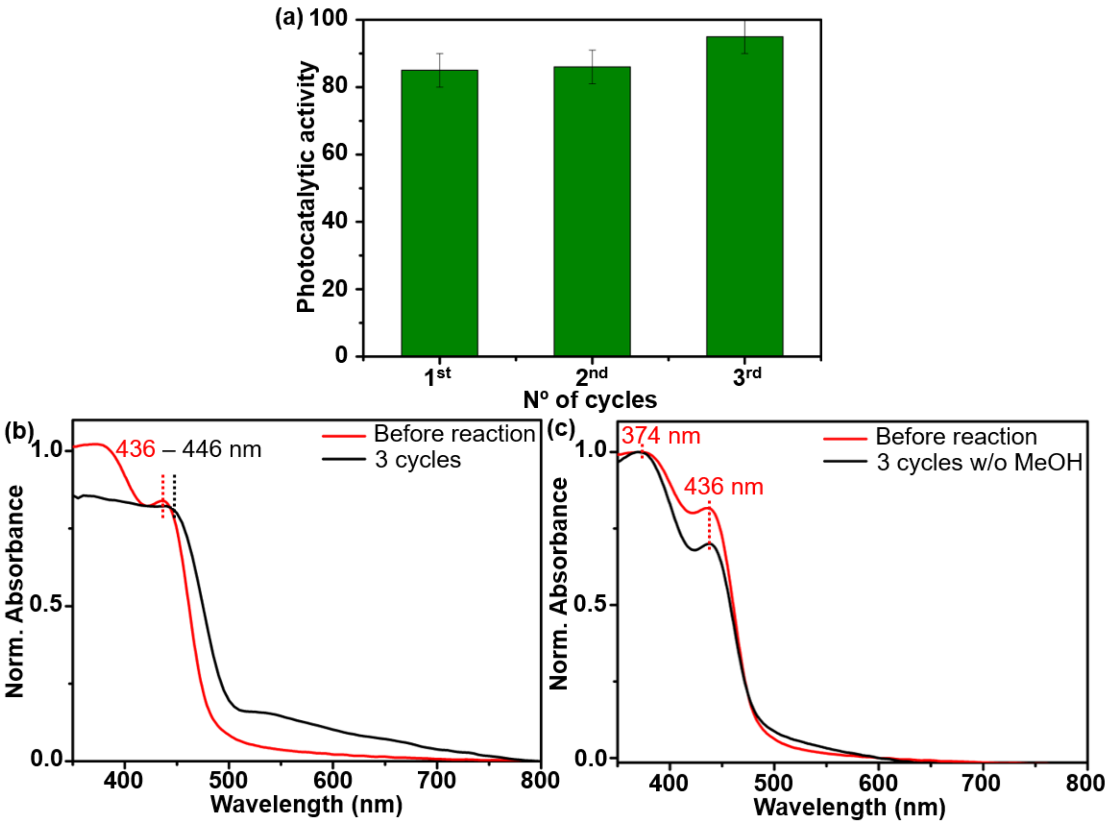

Figure S27. (a) Photocatalytic activity of the recycling experiments performed under standard conditions for 48 hours each cycle and triplicate. Optical absorption of the NCs before the reaction (red line) and after three cycles (black line) (b) with and (c) without methanol.

4. References

1. Ravi, V. K.; Swarnkar, A.; Chakraborty, R.; Nag, A., Excellent green but less impressive blue luminescence from $CsPbBr_3$ perovskite nanocubes and nanoplatelets. *Nanotechnology* **2016,** *27* (32), 325708.
2. Kun, S. V.; Lazarev, V. B.; Peresh, E. Y.; Kun, A. V.; Voroshilov, Y. V., Phase equilibria in RbBr-Sb(Bi)Br_3 systems and crystal structures of compounds of the type $A_3B_2C_9$ (AI-Rb, Cs; BV-Sb, Bi; CVII-Br, I). *Izvestiya Akademii Nauk SSSR, Neorganicheskie Materialy* **1993,** *29*, 410-413.
3. Jain, A.; Ong, S. P.; Hautier, G.; Chen, W.; Richards, W. D.; Dacek, S.; Cholia, S.; Gunter, D.; Skinner, D.; Ceder, G.; Persson, K. a., The Materials Project: A materials genome approach to accelerating materials innovation. *APL Materials* **2013,** *1*, 011002.
4. Monshi, A.; Foroughi, M. R.; Monshi, M. R., Modified Scherrer equation to estimate more accurately nano-crystallite size using XRD. *World Journal of Nano Science and Engineering* **2012,** *2* (3).
5. Luo, X.; Han, Y.; Chen, Z.; Li, Y.; Liang, G.; Liu, X.; Ding, T.; Nie, C.; Wang, M.; Castellano, F. N.; Wu, K., Mechanisms of triplet energy transfer across the inorganic nanocrystal/organic molecule interface. *Nature Communications* **2020,** *11* (1), 28.
6. Lambert, J. B.; Black, R. D. H.; Shaw, J. H.; Papay, J. J., Electrophilic addition of molecular bromine to a stereochemically defined cyclopropane. *The Journal of Organic Chemistry* **1970,** *35* (10), 3214-3216.
7. Byrnell, C. J. A.; Coombes, R. G.; Hart, L. S.; Whiting, M. C., The reaction of bromine with cyclohexene in carbon tetrachloride. Part 1. Reactions in the absence of hydrogen bromide; presence of a scavenger. *Journal of the Chemical Society, Perkin Transactions 2* **1983,** (8), 1079-1086.
8. Davis, H. S., The relatives rates of bromination of the olefins. *J. Am. Chem. Soc.* **1928,** *50* (10), 2769-2780.
9. Rosokha, S. V.; Stern, C. L.; Ritzert, J. T., Experimental and Computational Probes of the Nature of Halogen Bonding: Complexes of Bromine-Containing Molecules with Bromide Anions. *Chemistry – A European Journal* **2013,** *19* (27), 8774-8788.
10. Vasilyev, A. V.; Lindeman, S. V.; Kochi, J. K., Molecular structures of the metastable charge-transfer complexes of benzene (and toluene) with bromine as the pre-reactive intermediates in electrophilic aromatic bromination. *New J. Chem.* **2002,** *26* (5), 582-592.
11. Fukuzumi, S.; Kochi, J. K., Transition-state barrier for electrophilic reactions. Solvation of charge-transfer ion pairs as the unifying factor in alkene addition and aromatic substitution with bromine. *J. Am. Chem. Soc.* **1982,** *104* (26), 7599-7609.
12. Hammick, D. L.; Hutson, J. M.; Jenkins, G. I., The photochemical addition of bromine to bromobenzene in carbon tetrachloride solution. *J. Chem. Soc.* **1938,** (0), 1959-1964.
13. Podgoršek, A.; Stavber, S.; Zupan, M.; Iskra, J., Visible light induced ‘on water’ benzylic bromination with N-bromosuccinimide. *Tetrahedron Lett.* **2006,** *47* (7), 1097-1099.
